# Defect-Engineered Hydroxylated Mesoporous Spinel Oxides as Bifunctional Electrocatalysts for Oxygen Reduction and Evolution Reactions

**DOI:** 10.1021/acsami.2c00254

**Published:** 2022-05-13

**Authors:** Wanchai Deeloed, Tatiana Priamushko, Jakub Čížek, Songwut Suramitr, Freddy Kleitz

**Affiliations:** † Department of Inorganic Chemistry − Functional Materials, Faculty of Chemistry, University of Vienna, A-1090 Wien, Austria; ‡ Department of Chemistry, Faculty of Science, Kasetsart University, Bangkok 10900, Thailand; § Department of Low-Temperature Physics, Faculty of Mathematics and Physics, Charles University, V Holešovičkách 2, CZ-180 00 Praha 8, Czech Republic

**Keywords:** mesoporous materials, nanocasting, mixed metal spinel oxides, vacancy defects, bifunctional electrocatalyst, ORR, OER, PALS

## Abstract

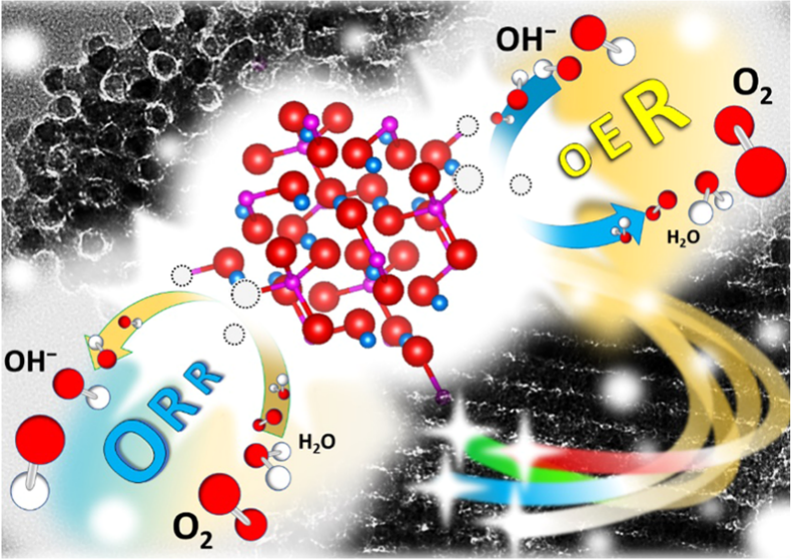

In this work, defect-rich ordered mesoporous spinel oxides, including CoCo_2_O_4_, NiCo_2_O_4_, and ZnCo_2_O_4_, were developed as bifunctional electrocatalysts toward oxygen reduction and evolution reactions (ORR and OER, respectively). The materials are synthesized via nanocasting and modified by chemical treatment with 0.1 M NaBH_4_ solution to enhance the defect concentration. The synthesized samples have metal and oxygen divacancies (V_Co_ + V_O_) as the primary defect sites, as indicated by positron annihilation lifetime spectroscopy (PALS). Cation substitution in the spinel structure induces a higher number of oxygen vacancies. The increased number of surface defects and the synergistic effect between two incorporated metals provide a high activity in both the OER and ORR in the case of NiCo_2_O_4_ and ZnCo_2_O_4_. Especially, ZnCo_2_O_4_ exhibits the highest OER/ORR activity. The defect engineering with 0.1 M NaBH_4_ solution results in a metal-hydroxylated surface (M-OH) and enhanced the catalytic activity for the post-treated metal oxides in the ORR and OER. This fundamental investigation of the defective structure of the mixed metal oxides offers some useful insights into further development of highly active electrocatalysts through defect engineering methods.

## Introduction

The increasing world population and the depletion of fossil fuel reserves have stimulated the utilization of renewable power sources. Storing and releasing electrical power through reversible electrochemical reactions are a key technological challenge on the way to green energy. Some promising energy storage devices, such as metal–air batteries (MABs) and regenerative fuel cells (RFCs), were studied within the last decades.^1−4^ These electrochemical devices use the water electrolysis principle, which is based on two reactions: oxygen evolution and reduction reactions (OER and ORR).^3^ The OER is one of the half-reactions in electrochemical water splitting. It uses energy from a renewable source, which then will be released back by carrying out the ORR.^5^ For both reactions, multistep electron transfer has been involved for one energy conversion cycle.^2,6^ The most efficient pathway for the ORR and OER under alkaline conditions is the direct 4 e^–^ pathway shown in eq 1(2)
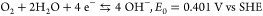
1

Meanwhile, the less-efficient pathway (also called 2 e^–^ pathway)
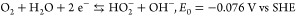
2followed by further reaction
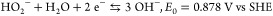
3also takes place.^2^ The presence of the 2 e^–^ pathway reduces the overall efficacy of the energy conversion device.^2^ Therefore, the rational design of bifunctional electrocatalysts capable of catalyzing both the OER and ORR through the 4 e^–^ pathway is crucial.

Noble metal-based catalysts, e.g., Pt/C, RuO_x_, and IrO_x_, are considered the most efficient ORR and OER catalysts.^3,5,7^ Recently, there were also efforts to develop other noble metal-based catalysts, e.g., PtSe_2_^8^ and RhSe_2_,^9^ for the hydrogen evolution reaction, which significantly contribute to the advancement of the water electrolysis technology. However, the scarcity and high prices of these precious elements have become an economic barrier to commercialization, resulting in the search for novel catalysts. A high abundance of the first-row transition metals associated with good stability of the oxides in harsh environments has stimulated the use of transition metal oxides (TMOs).^3−5,7^ Among them, cobalt oxide with a spinel structure, CoCo_2_O_4_ (or Co_3_O_4_), has gained attention due to its promising electrochemical performance.^10^ The presence of multivalent cobalt cations in octahedral (O_h_) and tetrahedral (T_d_) sites in the spinel structure determines the rich redox chemistry of the material.^11,12^ However, Co_3_O_4_ still suffers from drawbacks, including poor conductivity (10^–3^–10^–4^ S·cm^–1^) and the hazardous properties of cobalt,^13^ and concerns regarding the access and exploitation of the Co resources. Therefore, the idea of incorporation of foreign 3d elements, which are cheaper and more abundant, such as Mn, Fe, Ni, and Zn, has attracted great interest in this research area.^4,14−16^ The strategy is beneficial for the discovery of state-of-the-art multimetal oxide/(oxy)hydroxide catalysts for electrochemical catalysis owing to synergistic effects.^17−21^ Herein, two mixed metal cobalt-based spinel oxides, NiCo_2_O_4_ and ZnCo_2_O_4_, obtained by the partial substitution of cobalt by nickel and zinc, are considered. These spinel catalysts have been most commonly investigated owing to their remarkable redox chemistry, good durability, and low material cost.^22^ Moreover, both materials were recognized as excellent electrode materials for supercapacitors,^23^ lithium-ion batteries,^24^ and metal–air batteries.^25^

In addition to the chemical composition, the number and nature of the electrochemical catalytic sites are also essential for a highly reactive catalyst.^26^ Oxygen vacancies are known as the catalytic sites in cobalt-based oxides.^27,28^ To induce oxygen vacancies on the metal oxide surface, high-temperature reduction or some advanced techniques might be applied.^29,30^ Recently, Yan et al. have reported a facile chemical treatment using aqueous NaBH_4_ solution to introduce oxygen vacancies in NiCo_2_O_4_ nanowires.^23^ Similarly, several studies reported that NaBH_4_ treatment increased the number of oxygen vacancies in Co_3_O_4_, resulting in better water oxidation performance.^31,32^ Therefore, this demonstrates the benefit of using NaBH_4_ to create the oxygen vacancies on the surface of cobalt-based spinel oxides.

To obtain an optimal number of catalytic sites from NaBH_4_ treatment, the starting material should have a porous mesostructure providing a large specific surface area and a convenient flow for the chemical reactants. The synthesis of ordered mesoporous catalysts is performed to achieve this objective. Hard templating is a popular approach to obtain mesoporous TMOs with a controlled pore size distribution (PSD) and high specific surface area (SSA).^33−35^ Considering the reliability of the preparation technique, nanocasting shows some advantages to obtain the desired TMOs.^36−38^

Herein, we demonstrate the synthesis of mesoporous cobalt-based spinel oxides with nickel and zinc substitution followed by post-treatment with NaBH_4_, to vary the type and concentration of defects in the spinel structures. We establish the influence of the cation substitution and post-treatment with NaBH_4_ on the electrocatalytic behavior of these hydroxylated materials toward the OER and ORR. Furthermore, positron annihilation lifetime spectroscopy (PALS), a technique used mostly for bulk material analysis, was introduced as a potent characterization method of the metal oxide nanocasts for analyzing the defects in the crystal structure. By employing this method, we carried out in-depth qualitative and quantitative characterization of the introduced defects, which is usually achieved by the combination of other techniques such as X-ray photoelectron spectroscopy (XPS), Raman spectroscopy, high-angle annular dark-field imaging in scanning transmission electron microscopy (HAADF-STEM), or extended X-ray absorption fine structure (EXAFS), some of which require advanced and expensive equipment.^8,9,39−41^ The detailed study on surface functionality, defect sites, the number of electrons transferred (*n*), and material electrochemical impedance provides a new knowledge for the development of bifunctional highly efficient electrocatalysts.

## Experimental Section

### Preparation of a Highly Ordered Mesoporous Silica Template

The preparation of high-quality ordered mesoporous KIT-6 silica was performed by following the procedure reported by Kleitz et al.^42^ Typically, 5.13 g of triblock copolymer poly(ethylene glycol)-block-poly(propylene glycol)-block-poly(ethylene glycol) (Pluronic P123, EO_20_PO_70_EO_20_, MW = 5800, Sigma-Aldrich), as a structure-directing agent, was dissolved in acidic aqueous medium containing 9.93 g of hydrochloric acid (37 wt % HCl, VWR Chemicals, Germany) and 185.33 g of distilled water in a polypropylene (PP) bottle. After that, 5.13 g of *n*-butanol (99%, ThermoFisher (Kandel), Germany) was added at once to the mixture as a co-structure-directing agent. After stirring at 35 °C for at least 1 h, 11.03 g of tetraethyl orthosilicate (99% TEOS, Sigma-Aldrich, Germany) was added to the mixture at once while stirring, and stirring was continued for 24 h at 35 °C. Next, the reaction vessel was transferred to a hot-air oven (Binder, Germany). The siliceous mixture was kept under static conditions at 100 °C for 48 h. Afterward, the white precipitate was collected through filtration while hot, without washing, followed by drying at 100 °C for 2 h and at 140 °C overnight, sequentially. The template removal step was carried out by stirring the precursor powder in an acidic ethanolic solution, including 200 mL of ethanol (96% Brenntag, Austria) and 2 drops of concentrated HCl, at room temperature for 40 min, then filtered out, and dried at 70 °C. Finally, the powder was calcined at 550 °C for 3 h in air (heating rate ≈1 °C·min^–1^).

### Preparation of Highly Ordered Mesoporous Metal Oxide Spinel Replicas

Ordered mesoporous metal oxides were prepared by one-step impregnation nanocasting as reported by Yen et al.^43^ Co(NO_3_)_2_·6H_2_O, Ni(NO_3_)_2_·6H_2_O, and Zn(NO_3_)_2_·6H_2_O were used as the metal precursors and were supplied in analytical grade by ThermoFisher (Kandel), Germany. Typically, 2.5 g of metal nitrate was ground together with 1.0 g of KIT-6 silica powder in an agate mortar with *n*-hexane (10 mL, ThermoFisher, Germany) until dried. The silica template was outgassed (150 °C, overnight) before use. Then, the mixture was dispersed in 30 mL of *n*-hexane in a round-bottom flask and refluxed under vigorous stirring at 85 °C overnight. By doing so, the molten metal salts infiltrate the mesoporous network of the silica template, resulting in an inverse replication of the template structure. The infiltrated template was then filtered out, without washing, and dried at 70 °C. After that, an oxidative thermal conversion step was performed at 500 °C (heating rate ≈1 °C·min^–1^) in air for 5 h. The silica template was then chemically removed by dispersing the calcined powder in 2 M NaOH at 80 °C overnight twice. Then, the powder was thoroughly washed with distilled water and ethanol three times and dried at 70 °C in a hot-air oven. For the Ni- and Zn-substituted cobaltite spinels, the molar ratio between the cobalt nitrate and another metal nitrate of choice was equivalent to 2:1. The mesoporous CoCo_2_O_4_, NiCo_2_O_4_, and ZnCo_2_O_4_ spinel-structured catalysts are denoted as m-CCO, m-NCO, and m-ZCO, respectively.

### Chemical Treatment with NaBH_4_ Solution

NaBH_4_ powder (98%, ThermoFisher (Kandel), Germany) was used to prepare 0.1 M NaBH_4_ aqueous solution. For lab-scale synthesis, 250 mg of the prepared (mixed) metal oxide sample was dispersed in 12.5 mL of the freshly made 0.1 M NaBH_4_ solution in a closed PP centrifuged tube. The sealed tube containing the sample mixture was manually shaken with closed inspection, and the cap was frequently unscrewed for pressure rebalancing. The treatment was carried out for 1 h at room temperature. After the treatment, the sample powder was separated by centrifugation and washed two times with deionized water and ethanol and then dried at 70 °C in a hot-air oven. The mesoporous oxides after the post-treatment are named as m-CCO-NB, m-NCO-NB, and m-ZCO-NB.

### Material Characterization

Wide-angle powder X-ray diffraction (XRD) for phase identification was performed within 2θ ranging from 10 to 90° (scan speed = 0.01° s^–1^). The investigation was carried out using a powder diffractometer (PANalytical, EMPYREAN) in the reflection geometry, equipped with a Bragg-Brentano high-definition (BBHD) incident beam module, Cu Kα radiation source (45 kV, 40 mA), fixed-divergence slit (0.4354 mm), and PIXcel^3^ detector (Malvern PANalytical, U.K.). The low-angle XRD profile of the prepared catalysts was examined using the same diffractometer in the transmission mode using a focusing mirror incident beam module in the 2θ range of 0.5–4.0° (scan speed = 0.01° s^–1^). Inductively coupled plasma–mass spectrometry (ICP-MS) was carried out for elemental analysis, utilizing an Agilent ICP-MS 7800, coupled with an Agilent SPS 4 autosampler (number of replicates = 7, number of sweeps = 100). X-ray photoelectron spectra (XPS, Nexsa, Thermo-Scientific, MA) were collected using an Al Kα radiation source operating at 72 W and an integrated flood gun. A pass energy of 200 eV, the “standard lens mode”, the CAE analyzer mode, and an energy step size of 1 eV for the survey spectrum were used. The diameter of the X-ray beam was 400 μm. High-resolution spectra of C 1s, O 1s, Co 2p, Ni 2p, and Zn 2p were acquired with 50 passes at a pass energy of 50 eV and an energy step size of 0.1 eV. Routine XPS spectral analysis has been performed on Avantage software (Thermo Avantage v5.9922). The binding energy was calibrated using the C 1s peak (284.5 eV).

N_2_ physisorption measurements were carried out at −196 °C for the determination of the material porosity using an Anton Paar Quantatech Inc. iQ2 instrument (Boynton Beach, FL). The samples were outgassed under vacuum at 150 °C for 12 h before the measurement. ASiQwin 5.2 software (Anton Paar Quantatech Inc.) was used for data analysis. The Brunauer–Emmett–Teller specific surface area (*S*_BET_) was derived from data points within the relative pressure range of 0.05–0.3, whereas the total pore volume (with a pore size smaller than 40 nm) was calculated using Gurvich’s rule. The pore size distribution (PSD) was calculated by applying nonlocal density functional theory (NLDFT) with the kernel of metastable adsorption on the adsorption branch and the kernel of equilibrium desorption on the desorption branch, respectively, considering an amorphous SiO_2_ (oxide) surface and a cylindrical pore model.^44^

The mesoporous structure of the prepared catalysts was visualized by field-emission transmission electron microscopy (FETEM, JEOL-JEM-3100F, operating voltage 300 kV). Energy-dispersive X-ray spectroscopy (EDX) for chemical composition evaluation was performed on a scanning electron microscope (Zeiss Supra 55 VP, Faculty Center for Nano Structure Research, University of Vienna, Vienna, Austria) equipped with an EDX detector (Oxford Instruments).

Positron annihilation lifetime spectroscopy (PALS) was used to investigate the defects in the synthesized materials. A ^22^Na radioisotope with an activity of 1 MBq was used as a positron source. The positron source was deposited on a 7 μm-thick Kapton foil (DuPont) and was placed in the center of a small cylindrical chamber with a diameter of 6 mm and a height of 5 mm. Subsequently, the chamber was filled with the measured powder and closed. Dimensions of the chamber ensure that virtually, all positrons are thermalized inside the chamber and, thereby, annihilated in the studied powder. Positron lifetime (LT) measurements were carried out using a digital PL spectrometer.^45,46^ The spectrometer is equipped with BaF_2_ scintillators and Hamamatsu H3378 fast photomultipliers. Detector pulses are directly digitized using a couple of Acqiris DC 211 8-bit ultrafast digitizers with a sampling frequency of 4 GHz and stored in a PC. Analysis of digitized pulses and construction of the LT spectrum were performed offline using the so-called integral true constant fraction technique.^47^ The spectrometer exhibits a time resolution of 145 ps (FWHM of resolution function for ^22^Na). At least 10^7^ positron annihilation events were collected in LT spectra. Decomposition of LT spectra into individual components was performed using PLRF code version 19.^48^ The source contribution to LT spectra consisted of two weak components with lifetimes of ∼380 ps and ∼1.9 ns and the corresponding intensities of ∼14 and ∼1%. Coincidence Doppler broadening (CDB)^49^ studies were carried out using a digital spectrometer^50^ equipped with two high-purity Ge detectors. The energy resolution of the CDB spectrometer is 0.99 ± 0.02 keV at 511 keV. At least 10^8^ annihilation events were collected in each two-dimensional γ-ray energy spectrum, which was subsequently reduced into two one-dimensional cuts representing the resolution function of the spectrometer and the Doppler-broadened annihilation profile. CDB results are presented as ratio curves with respect to a well-annealed pure Co (99.99%) reference sample.

### Electrochemical Measurements

For the working electrode (WE) preparation, a glassy carbon rotating disk electrode (model RDE.GC50.S, Metrohm AG) was polished using 1.0 and 0.05 μm alumina colloidal suspensions (Beuhler) on a nonwoven polishing cloth (Beuhler), cleaned in water and acetone, and dried to obtain a mirror-finish GC surface before applying the catalyst ink. The ORR catalyst ink formula was adapted from the ink recipe, reported by Yuan et al.^51^ Typically, 5.0 mg of catalyst powder and 5.0 mg of carbon black (Super P conductive 99+% metal basis, ThermoFisher (Kandel), Germany) were dispersed in a mixture solution, containing nanopure water (700 μL), iso-propanol (250 μL, Sigma-Aldrich, Germany), and Nafion solution (50 μL, 5 wt % Nafion 117 solution, Aldrich), using ultrasonication for 1 h. A total of 10.0 μL of the as-prepared ink was applied on the GC surface to achieve a catalyst loading of ca. 0.25 mg·cm^–2^ and then directly irradiated using a tungsten lamp until dried. Platinum on graphitized carbon powder (Pt@C, 10 wt %, Aldrich) was used as a reference material and was prepared similarly to the metal oxide inks for the ORR. However, the carbon black additive was excluded from the Pt@C ink recipe. Note that only 5.00 μL of the ink, aiming for a Pt@C loading on the GC surface of ∼0.13 mg·cm^–2^, was applied. The OER ink was prepared by sonicating 5 mg of the catalyst without carbon in 750 μL of nanopure water, 250 μL of iso-propanol, and 50 μL of Nafion. Then, 5 μL of the homogeneous ink was drop-casted on the RDE surface aimimg for a loading of 0.12 mg·cm^–2^.

### Oxygen Evolution Reaction (OER)

OER catalytic activity was examined in a Teflon container with a standard three-electrode configuration. The GC-RDE electrode covered with a thin catalyst film was used as a working electrode (WE), whereas the Pt sheet (1 cm^2^, Metrohm) and hydrogen reference electrode (Hydroflex, Gaskatel) were employed as the counter electrode (CE) and reference electrode (RE), respectively. The standard WE RDE rotating speed was 2000 rpm. All investigations were performed in N_2_-saturated 1 M KOH aqueous solution (prepared using 85% KOH pellets, ThermoFisher (Kandel), Germany). The electrolyte was saturated with N_2_ before and during the measurements. The catalyst film was tested in the linear sweep voltammetry mode (LSV) in the potential range between 0.70 and 1.70 V vs RHE, using a scan speed of 10 mV·s^–1^.^38^ Cyclic voltammetry (CV) was performed at the scan rate of 50 mV·s^–1^ from 0.6 to 1.6 V vs RHE. Electrochemical double-layer capacitance (EDLC or *C*_dl_) was examined by performing CV in the non-faradaic region (1.00–1.10 V vs RHE) at different scan rates (20 to 180 mV·s^–1^). Electrochemical impedance spectroscopy (EIS) was carried out within the alternating current frequency ranging from 0.1 to 10^5^ Hz with an amplitude of 5 mV at an anodic polarization potential of 1.6 V vs RHE applied on the WE. EIS spectral fitting was performed with NOVA v. 2.1.3 software.

### Oxygen Reduction Reaction (ORR)

The catalytic activity of the materials in the ORR was evaluated in a similar three-electrode configuration. In this case, the Ag/AgCl (3.0 M KCl, Metrohm) electrode was used as a RE. The electrolyte was 0.1 M KOH aqueous solution (prepared from 90% KOH flakes, Sigma-Aldrich, France). The Ag/AgCl RE was calibrated against the reversible hydrogen electrode (RHE) to convert the voltage scale.

Linear sweep voltammetry (LSV) was performed in the potential window of 0.15–1.05 V vs RHE (potential sweeping rate = 10 mV·s^–1^) with a WE rotating speed of 1600 rpm. The temperature of the cell was kept at 24–25 °C during all the measurements. The ohmic drop was compensated for 90% using NOVA 2.1.3 software. The catalyst surface was activated by performing 20 CV cycles (0.05–1.05 V vs RHE, potential sweeping rate of 100 mV·s^–1^) in N_2_-saturated 0.1 M KOH electrolyte. The three-electrode cell was purged with O_2_ gas (99.999%) for at least 20 min before the ORR experiment. Assessment of *C*_dl_ in the ORR was similar to the method used in the OER.

The LSV experiment with different WE rotating speeds (400–2500 rpm) was carried out to assess the number of electrons transferred (*n*) by the Koutecký–Levich equation (eq 4) as follows^2,51^
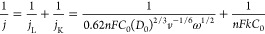
4where *j*, *j*_L_, and *j*_K_ represent the measured, diffusion-limiting, and kinetic-limiting current densities, respectively; *n* is the number of transferred electrons; *F* is the Faraday constant; *C*_0_ and *D*_0_ are the bulk concentration and the diffusion coefficient of O_2_ in O_2_-saturated 0.1 M KOH, respectively; *ν* is the kinematic electrolyte viscosity; ω is the working electrode rotation speed (rad·s^–1^); and *k* is the electron transfer rate constant.

Moreover, the accelerated durability test (ADT), including 1000 CV cycles (0.60–1.05 V vs RHE, a scan speed of 100 mV·s^–1^) and LSV (before and after the CV step) in O_2_-saturated 0.1 M KOH electrolyte, was performed to test the short-term stability of the materials. In addition to the experiments on the GC-RDE electrode, the development of an intermediate byproduct along the potential sweep was examined by rotating ring-disk electrode (RRDE) (model RRDE.GCPT.S, Metrohm AG) experiments with a procedure similar to the Koutecký–Levich analysis with the GC-RDE. The applied potential on the Pt ring for further byproduct oxidation was kept constant at 1.476 V vs RHE. For the RRDE experiments, the number of electrons transferred (*n*) and percentage of HO_2_^–^ production can be estimated from the ring current (*I*_R_) and disk current (*I*_D_) according to eqs 5 and 6(2,51)
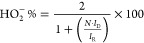
5
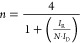
6where *N* represents the collection efficiency of the Pt ring = 0.249 in our case.

## Results and Discussion

### Physicochemical Characteristics of the Synthesized Materials

The porosity and mesostructure of the prepared KIT-6 silica were examined by performing N_2_ physisorption at −196 °C and low-angle XRD. The KIT-6 silica isotherm was identified as a type IV(a) sorption isotherm with a distinctive H1 hysteresis loop (Figure S1a), correlating with the previously published results.^42,52^ A narrow pore size distribution (PSD) with the center at 8.5 nm was obtained by NLDFT calculations from the desorption branch of the isotherm using the kernel of equilibrium desorption (Figure S1b). Moreover, the well-defined network mesopore structure with the cubic *Ia3d* pore symmetry was confirmed by the low-angle XRD profile in Figure S1c.^42^

**Figure 1:**
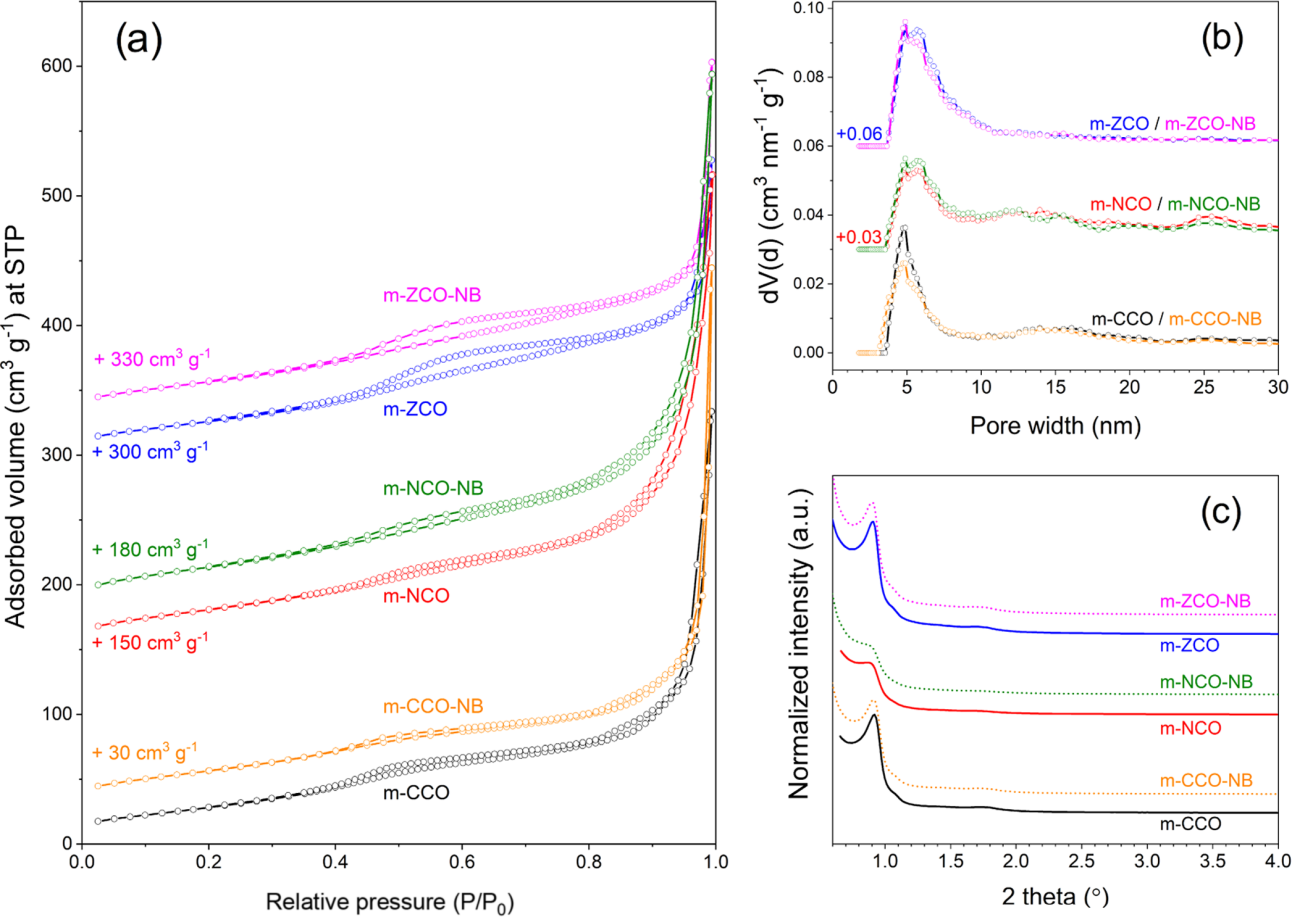
(a) N_2_ physisorption isotherms obtained at −196 °C, (b) NLDFT pore size distribution derived from the N_2_ adsorption branch, and (c) low-angle XRD patterns of the ordered mesoporous (mixed) metal oxides before and after the NaBH_4_ treatment.

N_2_ physisorption isotherms of the pristine metal oxides (m-CCO, m-NCO, and m-ZCO) exhibit a type IV(a) sorption isotherm associated with a mixed character of H2(b) and H3 hysteresis loops (Figure 1a).^52^ This result suggests complex porosity features of the replicas deriving from the KIT-6 silica template.^52^ The lower limit of the desorption branch at *P*/*P*_0_ of around 0.4 indicates a contribution of the cavitation-induced effect that affects the approximation of pore width.^44,52^ Therefore, a more reliable PSD should be derived from the adsorption branch of the isotherm using NLDFT and the kernel of metastable adsorption.^44^ As a result, the PSD with the mode value at around 5 nm was obtained (Figure 1b). The specific surface area and the total pore volume, estimated by Brunauer–Emmett–Teller theory (*S*_BET_) and Gurvich’s rule, respectively, are similar across the sample series (Table 1), even though the shape of the hysteresis loop of NaBH_4_-treated samples slightly deviates from that of the untreated samples. These results are in line with published data, suggesting the good quality of the replicas.^53^

**Table 1:** Porosity Characteristics of the KIT-6 Silica and the Mesoporous Mixed Metal Oxides as Examined by N_2_ Sorption Experiments at −196 °C

sample	*S*_BET_ (m^2^·g^–1^)	NLDFT pore size (nm)	total pore volume^a^ (cm^3^·g^–1^)
KIT-6 (100 °C)	824	8.5	1.3
m-CCO	111	4.7	0.3
m-CCO-NB	107	4.9	0.2
m-NCO	121	4.9	0.3
m-NCO-NB	131	4.9	0.3
m-ZCO	105	4.9	0.2
m-ZCO-NB	108	4.9	0.2

a Total pore volume was estimated from pores smaller than 40 nm (*P*/*P*_0_ ≈ 0.95).

The ordered mesoporous structure of the synthesized metal oxides was confirmed by low-angle XRD (Figure 1c). Each sample exhibits a prominent diffraction peak and a shoulder peak at a 2θ of 0.9 and 1.1°, corresponding to 221 and 220 reflections of cubic *Ia3d* pore symmetry, respectively.^36,54^ In the nickel-containing catalyst (m-NCO and m-NCO-NB) cases, the diffraction intensity of the 221 and 220 reflections is less pronounced than that of the other samples, suggesting a less-effective mesostructure replication when mixed metal salts containing Ni^2+^ ions are used. However, the diffraction profile of these samples is still in line with that of the metal oxides synthesized via nanocasting.^38,53^ N_2_ sorption and low-angle XRD results demonstrate that even though a minor change on the hysteresis loop was spotted, the parent’s mesostructure and porosity were well-preserved in the NaBH_4_-treated samples (m-CCO-NB, m-NCO-NB, and m-ZCO-NB, Figure 1). It confirms that the NaBH_4_ treatment does not affect strongly the mesostructure of the metal oxides.

**Figure 2:**
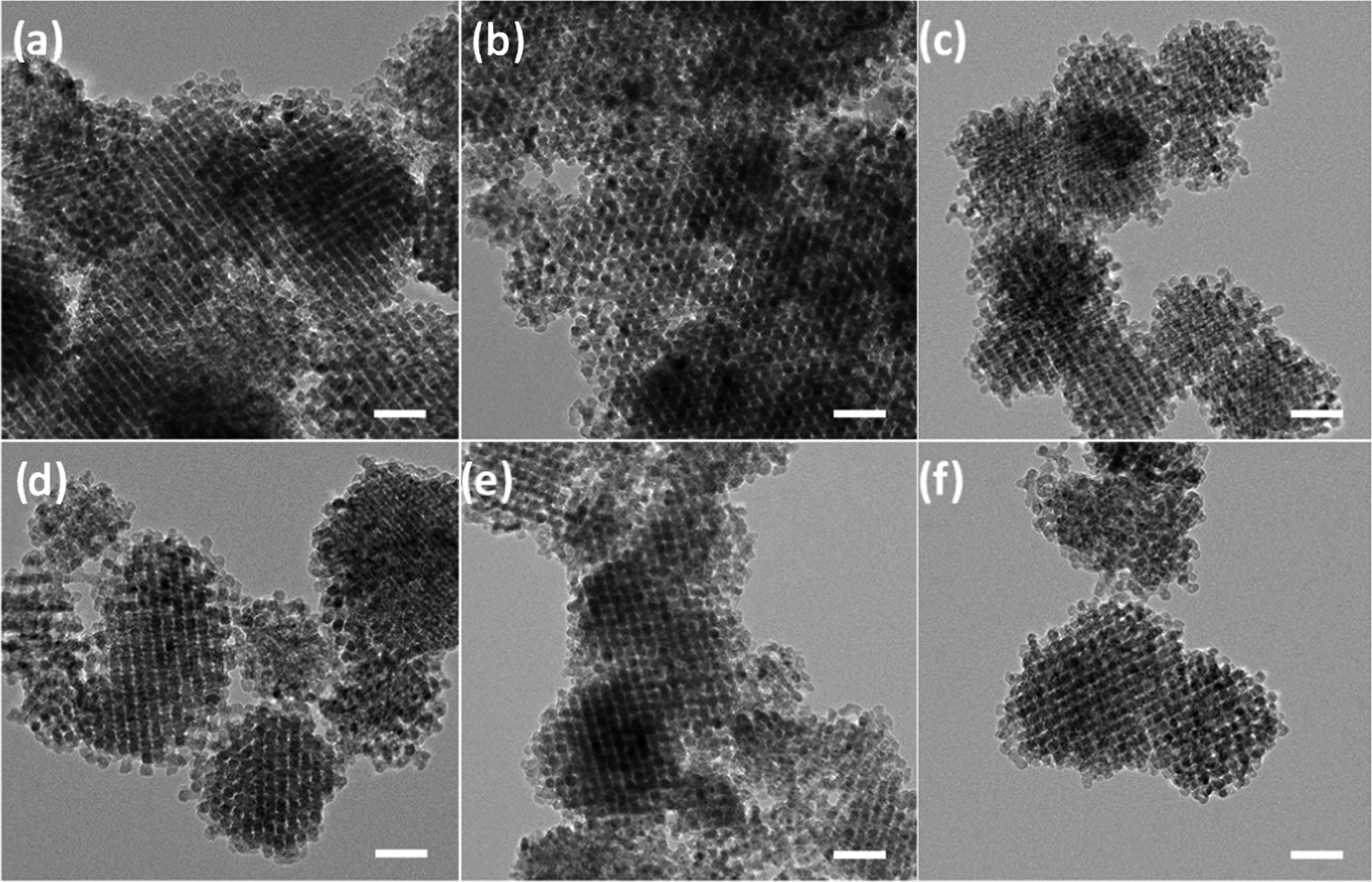
TEM images of the ordered mesoporous metal oxides: (a) m-CCO, (b) m-NCO, (c) m-ZCO, (d) m-CCO-NB, (e) m-NCO-NB, and (f) m-ZCO-NB. Scale bars are 50 nm.

The nanoscale porosity features of the synthesized materials were visualized by transmission electron microscopy (TEM). The TEM images (Figure 2a–f) reveal the ordered mesoporous network of the metal oxides. The catalysts mostly appear as spherically shaped particles. There is a broad distribution in the size of the particles, ranging from several hundred nanometers up to a few microns, which is typical for the replicas of the KIT-6 template.^38,53,55^

**Figure 3:**
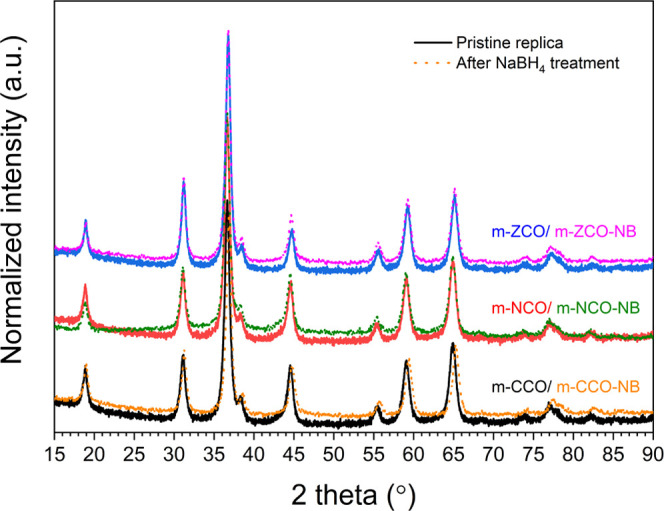
Wide-angle XRD patterns of the mixed metal oxides before (solid line) and after the NaBH_4_ treatment (dotted line).

Wide-angle XRD was used to identify the materials’ crystalline phases (Figure 3). All samples exhibit an XRD pattern, which corresponds to the crystalline cubic spinel phase (space group *Fd*3̅*m*). The observed reflections at a 2θ of 19.0, 31.3, 36.9, 38.5, 44.8, 55.7, 59.4, and 65.2° are assigned to the Bragg’s angles of reflections of (111), (220), (311), (222), (400), (422), (511), and (440) planes (PDF #98-006-9365), respectively. Most samples show no sign of a second phase, except m-NCO, which shows an additional peak at 43.7°, indicating the presence of an impurity—NiO phase (PDF #98-006-1318). All NaBH_4_-treated samples exhibit XRD patterns identical to the pristine ones, implying no substantial effect of the treatment on material bulk crystallinity. Rietveld refinement of the wide-angle XRD patterns was performed to analyze the spinel structures. The results in Figure S2 and Tables S1 and S2 confirm the purity of the crystalline spinel phase in most samples. For the nickel-containing oxides, the refinement suggests a small proportion of an emerged NiO phase, ca. 6–7 wt %. The size of the crystalline spinel domain of ca. 8–10 nm (Table S1) is comparable to the pore size of the KIT-6 template, as reported in Table 1. Furthermore, the result obtained by the refinement in Table S2 also suggests the metal vacancy defects.

Energy-dispersive X-ray spectroscopy (EDX) and inductively coupled plasma–mass spectrometry (ICP-MS) were performed for the elemental analysis. The results of spot-/area-EDX and ICP-MS are summarized in Tables S3–S5. For the nickel-containing catalysts (m-NCO and m-NCO-NB), the nominal Ni to Co ratio (around 0.5) perfectly matches with the one obtained from the analyses. However, for the zinc-containing catalysts (m-ZCO and m-ZCO-NB), the evaluation revealed a deficiency in Zn, according to a Zn to Co ratio of around 0.4. Hence, the chemical formula of the zinc-containing samples in this study is assumed to be Zn_0.85_Co_2.15_O_4_. In addition to this, the amounts of other possible elements (e.g., boron, sodium, and silicon) were negligible.

Due to the important role of oxygen vacancies in electrocatalytic processes, the described synthesis protocol was designed for maximizing the concentration of surface defects. Here, cation substitution and surface post-treatment were used for this purpose, and their effect was studied by PALS and XPS. A model of bulk Co_3_O_4_ with a perfect cubic spinel structure (Figure S3) was used to calculate the lifetime of free positrons delocalized in the perfect lattice and positrons trapped at specific defect sites. Ab initio calculations of positron lifetimes were performed using density functional theory (DFT) within the so-called standard scheme.^56^ It appears that the calculated lifetime of free positrons delocalized in the perfect Co_3_O_4_ lattice (bulk lifetime) is τ_B_ = 145.5 ps (Table S6). Oxygen vacancy (V_O_) is only a shallow trap characterized by a positron lifetime of 147.8 ps, which is only slightly higher than τ_B_. In contrast, the cobalt vacancies (V_Co_) appear to be the deep traps. The cubic spinel structure of Co_3_O_4_ consists of two crystallographically nonequivalent sites of Co ions denoted as Co-1 and Co-2 in Figure S3. It means that there are two kinds of Co vacancies related to the missing Co atom in the Co-1 site (V_Co-1_) or the Co-2 site (V_Co-2_). Calculated lifetimes of positrons trapped in V_Co-1_ and V_Co-2_ are 174.8 and 217.6 ps, respectively. In addition, Co+O divacancy defects were also considered, and they are characterized by longer lifetimes, namely 200.3 and 249.7 ps for V_Co-1_ + V_O_ and V_Co-2_ + V_O_, respectively. The higher the number of vacancies contained in the defect, the larger the open volumes and the longer the positron lifetime.

**Figure 4:**
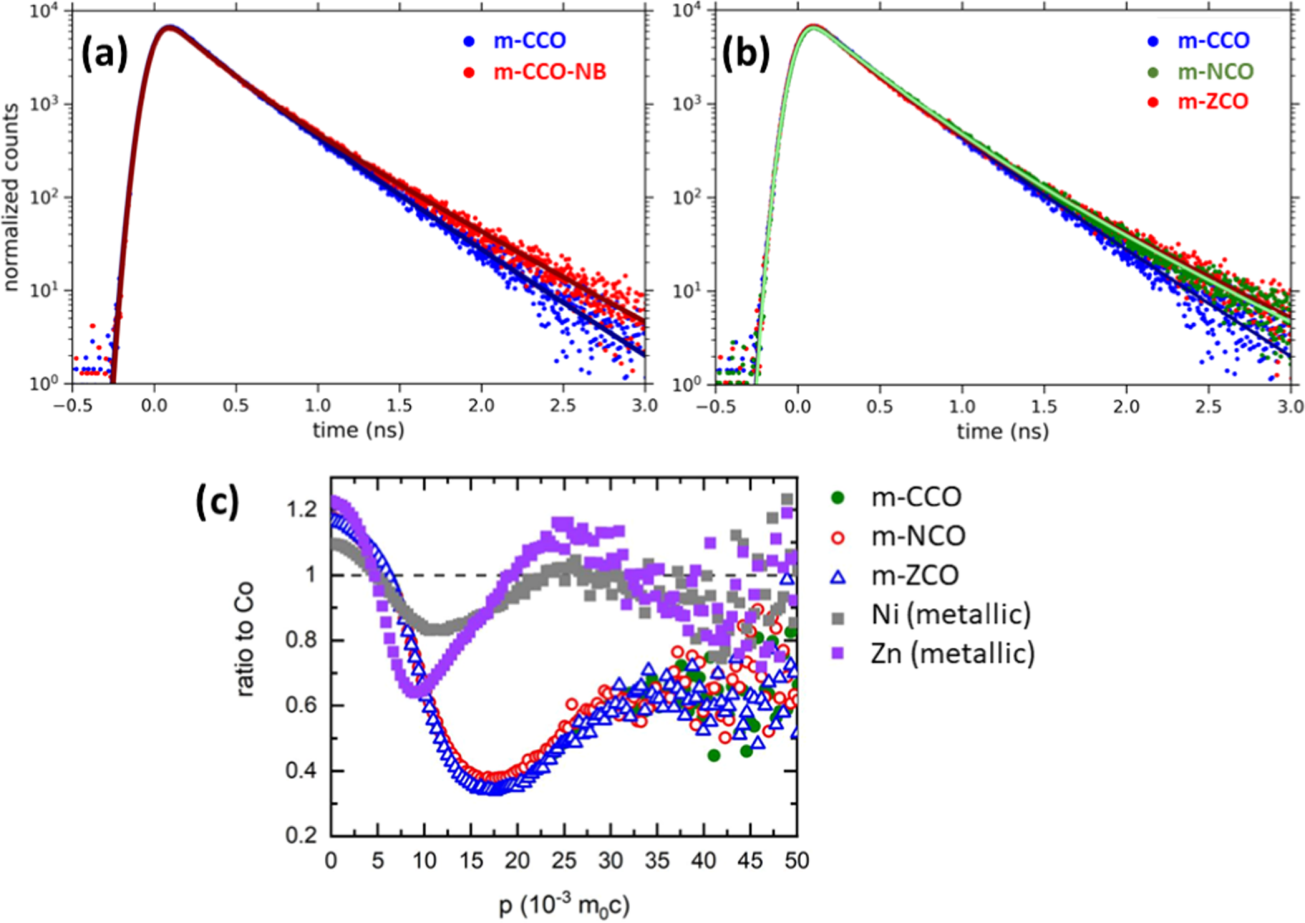
Positron lifetime spectra for (a) m-CCO and m-CCO-NB samples and (b) nontreated samples: m-CCO, m-NCO, and m-ZCO. Solid lines represent fits of spectra. (c) CDB ratio curves (related to pure metallic cobalt reference, dashed line) for nontreated samples.

Figure 4a shows the positron lifetime spectra of the m-CCO and m-CCO-NB samples. Both spectra are well-described by two components with lifetimes τ_1_ and τ_2_, as listed in Table S7. Significantly, both τ_1_ and τ_2_ lifetimes are higher than the theoretical positron lifetime in a perfect Co_3_O_4_ lattice, τ_B_ = 145.5 ps (Table S6). Moreover, the mean diffusion length of a thermalized positron is in the order of 100 nm, about 10 times the size of the spinel nanocrystal (ca. 8–10 nm) as determined by XRD.^57^ Therefore, it testifies that almost all positrons should be annihilated in the vacancy-type defects at the grain boundaries. For the m-CCO sample, the first component with the lifetime τ_1_ ≈ 240 ps (Table S7) should refer to the positrons trapped in the defect sites at the inter-grain boundaries, with an open volume comparable to V_Co-2_ + V_O_ divacancies. Meanwhile, the second component with a lifetime τ_2_ of 390 ps indicates a contribution of positrons trapped at defect sites on particle surfaces with larger open volumes. As a complement, the increased lifetime τ_2_ of 455 ps of the m-CCO-NB sample (Table S7) confirmed the sensitiveness of τ_2_ for the NaBH_4_ treatment. Furthermore, the NaBH_4_ treatment resulted in the increased intensity *I*_1_ of the first component, indicating an enhanced concentration of the defects at the inter-grain boundaries.

Figure 4b shows a comparison of positron lifetime spectra for all non-NaBH_4_-treated samples. Compared to m-CCO, the m-NCO and m-ZCO samples contain surface vacancy defects with a larger open volume, characterized by the longer τ_1_ and τ_2_ lifetimes, respectively (Table S7). In addition, the concentration of the surface defects at the inter-grain boundaries is enhanced for the m-NCO and m-ZCO samples as seen from a dominant contribution of *I*_1_ up to ca. 67% (Table S7). In addition, the effect of NaBH_4_ treatment on m-NCO and m-ZCO samples is similar to the one of m-CCO. The treatment usually enlarged the defect open volume (longer τ_1_ and τ_2_ lifetimes) and significantly increased the concentration of defects at the inter-grain boundaries (higher *I*_1_ contribution).

The increase in positron lifetime by nickel and zinc substitution might indicate the other positron trapped state that correlates with the material component, e.g., vacancies coupled with the substituents. To clarify this, CDB spectroscopy, which provides information about the local chemical environment of defects, was employed. CDB ratio curves with respect to the metallic cobalt reference for non-NaBH_4_-treated samples and ratio curves for the metallic nickel and zinc references are plotted in Figure 4c. Note that the CDB ratio curve for metallic cobalt is a constant equaling unity since all ratio curves were related to the cobalt reference. It was found that the CDB ratio curves for Ni- and Zn-substituted samples (m-NCO and m-ZCO) are very similar to the ratio curve for the unsubstituted Co_3_O_4_ sample (m-CCO). Since the m-CCO sample consists of Co and O, positrons can be annihilated only in the vicinity of either Co or O ions. The CDB ratio curves are related to Co; thus, any difference of the CDB curve for the m-CCO sample from constant is caused by the contribution of positrons annihilated in the vicinity of O ions. As seen in Figure 4c, the similarity of CDB ratio curves among m-NCO, m-ZCO, and m-CCO samples testifies that the segregation of nickel or zinc cations at open volume defects is insignificant. In other words, neither the nickel nor zinc cations are coupled with these defect sites. Hence, the increased lifetimes τ_1_ and τ_2_ in m-NCO and m-ZCO are not connected with the segregation of substituents at grain boundaries but rather with the change of V_Co-2_ + V_O_ into V_Co-2_ + *n*V_O_ (2 ≤ *n* ≤ 4). In other words, the result suggests a higher number of oxygen vacancies in the surface defect sites in the nickel- and zinc-containing samples.

**Figure 5:**
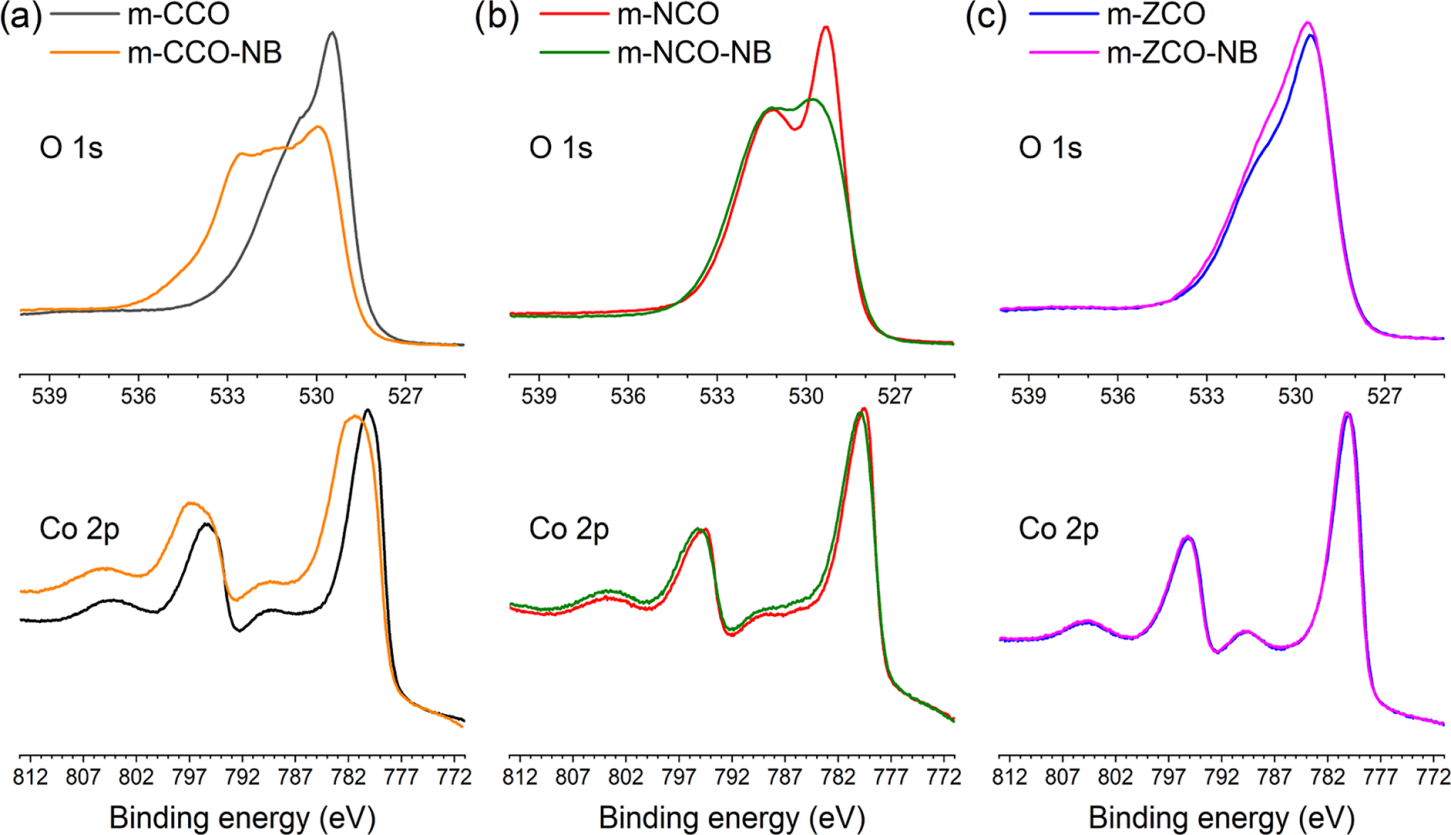
XPS spectra of O 1s and Co 2p of (a) m-CCO and m-CCO-NB, (b) m-NCO and m-NCO-NB, and (c) m-ZCO and m-ZCO-NB samples.

Apart from bulk characterization, the study of the material surface was carried out by XPS. The results in Figure 5 illustrate the comparative plots of the O 1s and Co 2p core shell XPS signals of each sample. As seen in Figure 5a, the NaBH_4_ treatment significantly affects O 1s and Co 2p states of m-CCO. Three native oxygen species, including the spinel-lattice oxygen (O^2–^) (O1, 529.4 eV), metal-hydroxyl oxygen (M-OH) (O2, 530.5 eV), and oxygen vacancy defect (O3, 531.1 eV), were indicated for the m-CCO and m-CCO-NB samples (see Figure S4 and Table S8).^39−41^ It appears that the contribution of O1 species (lattice O^2–^) decreased, while the contribution of O2 (M-OH) has distinctively increased through the NaBH_4_ treatment (Figure S4b). Moreover, oxygen in new environments, i.e., chemisorbed water (O4 at 532.8 eV and O5 at 534.5 eV) was detected on the m-CCO-NB.^32,58−60^ In addition to the O 1s core shell, an analysis of the Co 2p core level of m-CCO-NB in Figure 5a confirmed the presence of Co-OH species by the XPS contribution at 782.5 eV (Table S8).^59^ Hence, the XPS results suggest that the role of the diluted NaBH_4_ solution is to not only create a higher number of defects on the surface but also partially reduce the metal oxide surface (M-O-M) toward the metal-hydroxylated surface (M-OH). A similar effect of NaBH_4_ treatment was observed from the m-NCO-NB and m-ZCO-NB samples by analyzing their O 1s spectra (Figures 5b,c, S5, and S6 and Tables S9 and S10). However, it seems that a lower effectiveness of the NaBH_4_ treatment on surface modification was obtained through the nickel and zinc substitution (Figure 5b,c).

According to the results of in-depth characterization, the synthesized mesoporous oxides exhibit metal and oxygen divacancy defects (V_Co-2_ + V_O_) on the crystal grain boundaries. This is in line with the Rietveld refinement that indicated the deficiency of metal cations (Table S2). The incorporation of nickel and zinc significantly enhanced the concentration of oxygen vacancies. Meanwhile, the NaBH_4_ treatment provided a partially reduced metal oxide surface and enlarged the concentration of surface vacancies, as evident from the increase in the positron lifetimes τ_1_ and τ_2_. The defect sites corresponding to the lifetime τ_2_, which are located on the surface of the oxide particles, were more sensitive to the NaBH_4_ treatment. In contrast, the defect sites corresponding to the lifetime τ_1_, located at the inter-grain boundaries, were more difficult to react with NaBH_4_. Since the escape depth of X-ray photoelectron is in the range of a few nanometers, determining the defect sites at the inter-grain boundary is more challenging by XPS. Therefore, a noticeable change of O 1s spectra in Figure 5, occurring in accord with the change of the lifetime τ_2_ as reported in Table S7, could correspond to change of the surface of oxide particles where defects characterized by the lifetime τ_2_ are located. The XPS spectra likely do not include the information about inter-grain boundaries correlated with the lifetime τ_1_.

Consequently, the results suggest that the post-treatment using a diluted NaBH_4_ solution leads to the hydroxylation of the surface of the spinel material. In comparison, Yan et al.,^23^ Wei et al.,^32^ and Ortiz-Quiñonez et al.^59^ employed a similar strategy on other types of materials and found comparable results. Hence, this facile chemical treatment provides an excellent means to engineer the defects and produce OH-rich metal oxide surfaces.

### Catalytic Activity in the OER

**Figure 6:**
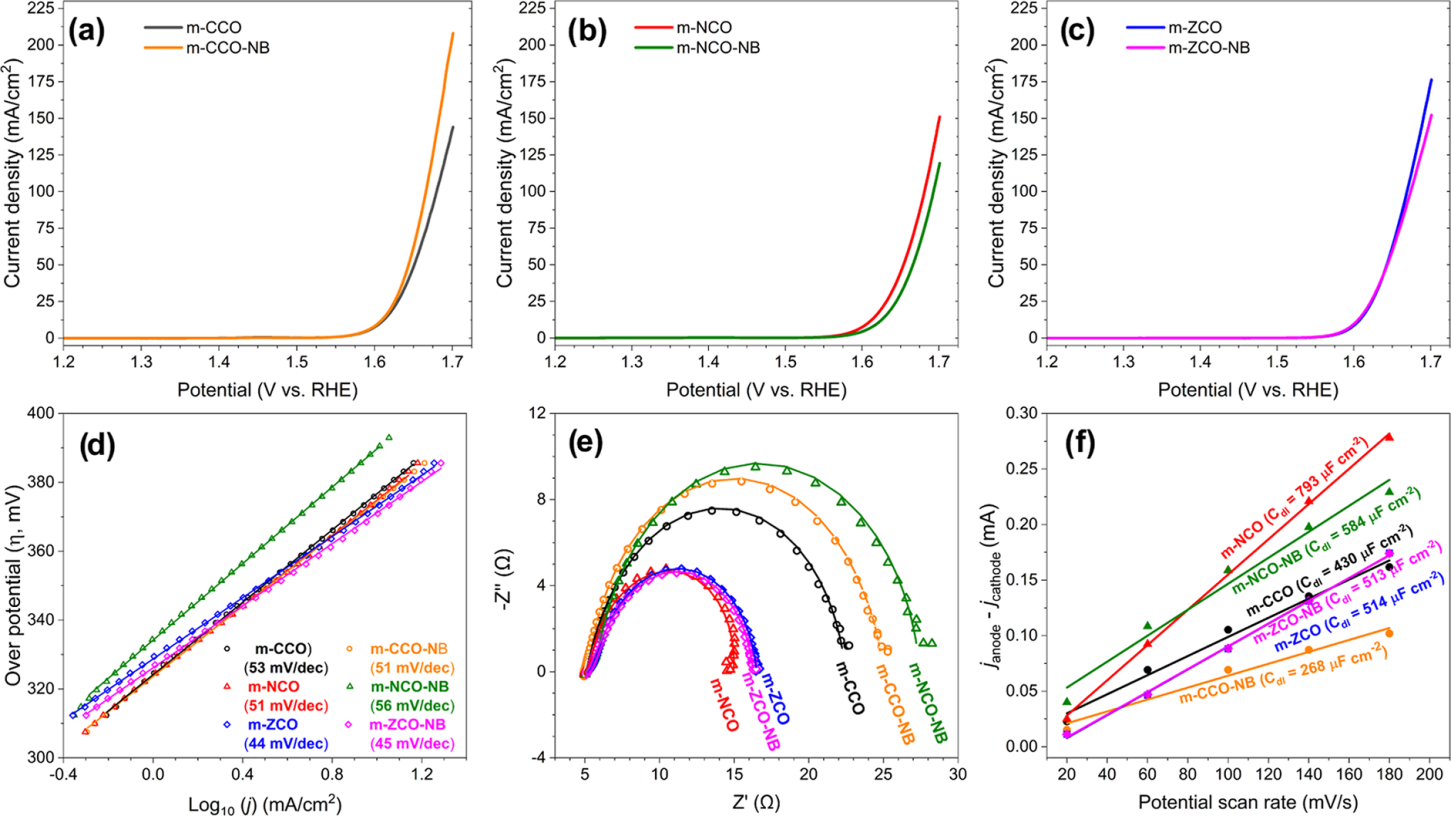
OER LSV curves of (a) m-CCO and m-CCO-NB, (b) m-NCO and m-NCO-NB, and (c) m-ZCO and m-ZCO-NB catalysts. (d) Tafel slopes, (e) Nyquist plots, and (f) double-layered capacitance (*C*_dl_) of every prepared sample in the OER experiment.

The catalytic performance of the prepared catalysts for the OER was examined in N_2_-saturated 1 M KOH solution at a scan rate of 10 mV·s^–1^ and a WE rotating speed of 2000 rpm. The results presented in Figure 6a–c show the responsive OER-LSV curves of the prepared catalysts. An active OER catalyst should reach the benchmark current density (*j*, 10 mA·cm^–2^) at the overpotential (η) closest to a thermodynamic potential for water splitting, 1.23 V vs RHE.^10^ It was found that the m-CCO sample reaches 10 mA·cm^–2^ at the overpotential (η) of 378 mV, which is similar to that of the previously reported ordered mesoporous Co_3_O_4_ sample^61^ (OM Co_3_O_4_, see Table S11 for a comparison of various electrocatalysts), and demonstrates better OER performance than that of the commercial Co_3_O_4_ because of its higher *S*_BET_. For the mixed metal oxides, the m-NCO reaches the benchmark current density at the same overpotential as the m-CCO sample. Meanwhile, the m-ZCO sample seems slightly more reactive than the other tested catalysts by reaching 10 mA·cm^–2^ at the overpotential of 373 mV. Interestingly, at the potential of 1.7 V vs RHE, the m-NCO and m-ZCO samples exhibit the current density of 151 and 176 mA·cm^–2^, respectively, which is greater than that of the m-CCO sample (144 mA·cm^–2^). In Figure 6d, the Tafel slopes of the m-NCO (51 mV·dec^–1^) and m-ZCO (44 mV·dec^–1^) samples are also lower than that of the m-CCO sample (53 mV·dec^–1^), indicating a better catalyst’s surface kinetics.^62^

Cyclic voltammetry at the non-faradaic region (1.00–1.10 V vs RHE, at different scan rates of 20–180 mV·s^–1^) has been carried out to evaluate the double-layered capacitance (*C*_dl_) and electrochemical surface area (ECSA) of the prepared catalysts. The resulting cyclic voltammograms for evaluating *C*_dl_ and ECSA are plotted in Figure S7, together with the analyzed ECSA value reported in Figure 6f and Table S11. The m-NCO and m-ZCO samples show significantly improved ECSA values of almost 85 and 20%, respectively, compared with the m-CCO catalyst (Table S11). Therefore, these results indicate the benefit of Ni and Zn substitution in the Co_3_O_4_ material to provide more catalytic sites, which is in good agreement with the discussion on the material defects as detailed above. Furthermore, this higher initial activity of the mixed metal oxides compared to that of m-CCO may be assigned to the alteration of the electronic structure upon strong electronic interaction between metals.

Cyclic voltammetry measurement (0.60–1.60 V vs RHE) revealed the characteristic anodic (A1) and cathodic peaks (A2), which can be seen for every sample between 1.4–1.5 V vs RHE (Figure S8). These A1/A2 redox-coupled peaks characterized the oxidation and reduction of Co^3+/4+^ species.^63−65^ Other redox-coupled peaks (A3 and A4), which were found at 1.1–1.2 V vs RHE (Figure S8a,d) in the m-CCO samples, indicated the oxidation and reduction of Co^2+/3+^ species as well.^65^ As expected, the later redox-coupled peaks were absent from the m-ZCO sample (Figure S8c) because of a substitution of Co^2+^ in the spinel structure by the Zn^2+^ species. In addition to these, responsive current owing to Ni^2+/3+^ oxidation (B1, ca. 1.30 V vs RHE) and reduction (B2, ca. 1.28 V vs RHE) was detected for the m-NCO sample (Figure S8b).^66^ The Ni redox-coupled peaks were more pronounced in the mixed metal oxide after NaBH_4_ treatment, which might point out the presence of a higher number of Ni^2+^ rather than Ni^2+/3+^. Furthermore, in the case of treated samples, the Co^2+^ oxidation peak is more pronounced, which might be assigned to the partial reduction of the Co species on the surface of these samples upon the NaBH_4_ treatment.

Electrochemical impedance spectroscopy (EIS) was employed to describe the reactivity of each prepared catalyst. Figure 6e illustrates Nyquist plots, which were recorded from each catalyst in the OER experiment. The results were further fitted with the model of the equivalent circuit model proposed by Bredar et al.^67^ (Figure S10). The simulated Nyquist plots are presented together with the measured ones in Figure S9. An excellent agreement between the theoretical and experimental impedance spectra was obtained, indicating the reliable fitting parameters reported in Table S12. Herein, the *R*_CT_ parameter imitates the impedance corresponding to the faradaic process’s charge transfer resistance of catalyst active sites. In comparison with m-CCO, nickel and zinc substitution positively affects the metal oxide catalyst by lowering *R*_CT_ values (Table S12). It is clearly seen that the samples are affected by the post-treatment in different ways. The resistance of the m-ZCO catalyst did not change with the treatment, which is in line with almost no change in the onset and overpotential. In the case of m-CCO and m-NCO, it was observed that the NaBH_4_ treatment generally increased *R*_CT_ (Table S12). This might be explained by the presence of a higher amount of reduced species (higher number of Ni^2+^ and Co^2+^ instead of Co^3+^ and Ni^3+^) on the surface of the catalysts and their lower reactivity. This is in line with the redox peaks obtained via CV (see the discussion given above). Moreover, the NaBH_4_ treatment generally reduced the ECSA of most OER catalysts, except only for the m-ZCO-NB case. This might also explain the enhanced charge transfer resistance of the m-CCO-NB and m-NCO-NB. It was observed that the treatment seems to be unsuitable for the nickel-containing sample since it significantly increased the Tafel slope, overpotential at 10 mA·cm^–2^, and reduced current density at 1.7 V vs RHE of the m-NCO-NB sample (Table S11). Meanwhile, a less negative effect from the NaBH_4_ treatment was observed on the m-ZCO-NB. While the Tafel slope and ECSA value have been maintained in this sample case, the decline of current density at 1.7 V vs RHE was obtained. In contrast, post-treated single-metal oxide m-CCO-NB provides the highest current density at 1.7 V vs RHE of 208 mA·cm^–2^, even though the ECSA value was reduced to 6.70 cm^2^. This suggests that although the treatment reduced the number of active sites on the surface of the catalysts, it enhanced their activity. Moreover, the stability of the m-CCO-NB catalyst analyzed by chronopotentiometry (Figure S11) is excellent. However, further stability tests are needed to ensure the durability of such materials.

To evaluate the intrinsic activity of the oxides, we normalized the collected current density by either ECSA or specific surface area (*S*_BET_). Figure S12 illustrates the normalized current densities of each catalyst. Since the prepared samples have a similar *S*_BET_ value, only the ECSA-normalized current density can deduce the variation in the activity of materials’ catalytic sites. It was found that the m-ZCO and m-ZCO-NB samples provide ECSA-normalized current density similar to the m-CCO sample. On the other hand, the lower ECSA-normalized current density suggested a less-active catalytic site in m-NCO and m-NCO-NB catalysts. Nevertheless, as a trade-off for the catalyst possessing a moderate intrinsic activity, the Ni substitution offers another advantage by providing a large additional ECSA to those samples, which is almost twice the value of the m-CCO catalyst according to the results presented in Table S13. Therefore, a high current density of m-NCO and m-NCO-NB in Figure 6b might indeed be derived from a high ECSA of the sample. The result highlights an advantage of utilizing zinc rather than nickel to substitute cobalt in the spinel oxide structure for OER catalysis. Apart from this, the high intrinsic activity of m-CCO-NB has been illustrated by the ECSA-normalized current density as well. Even though the smallest ECSA of the m-CCO-NB catalyst was obtained after NaBH_4_ treatment, the remaining catalytic sites are highly capable of producing the highest current density. Hence, optimization of these highly active catalytic sites will be beneficial for further improvement of Co_3_O_4_ catalysts.

### Catalytic Activity in the ORR

**Figure 7:**
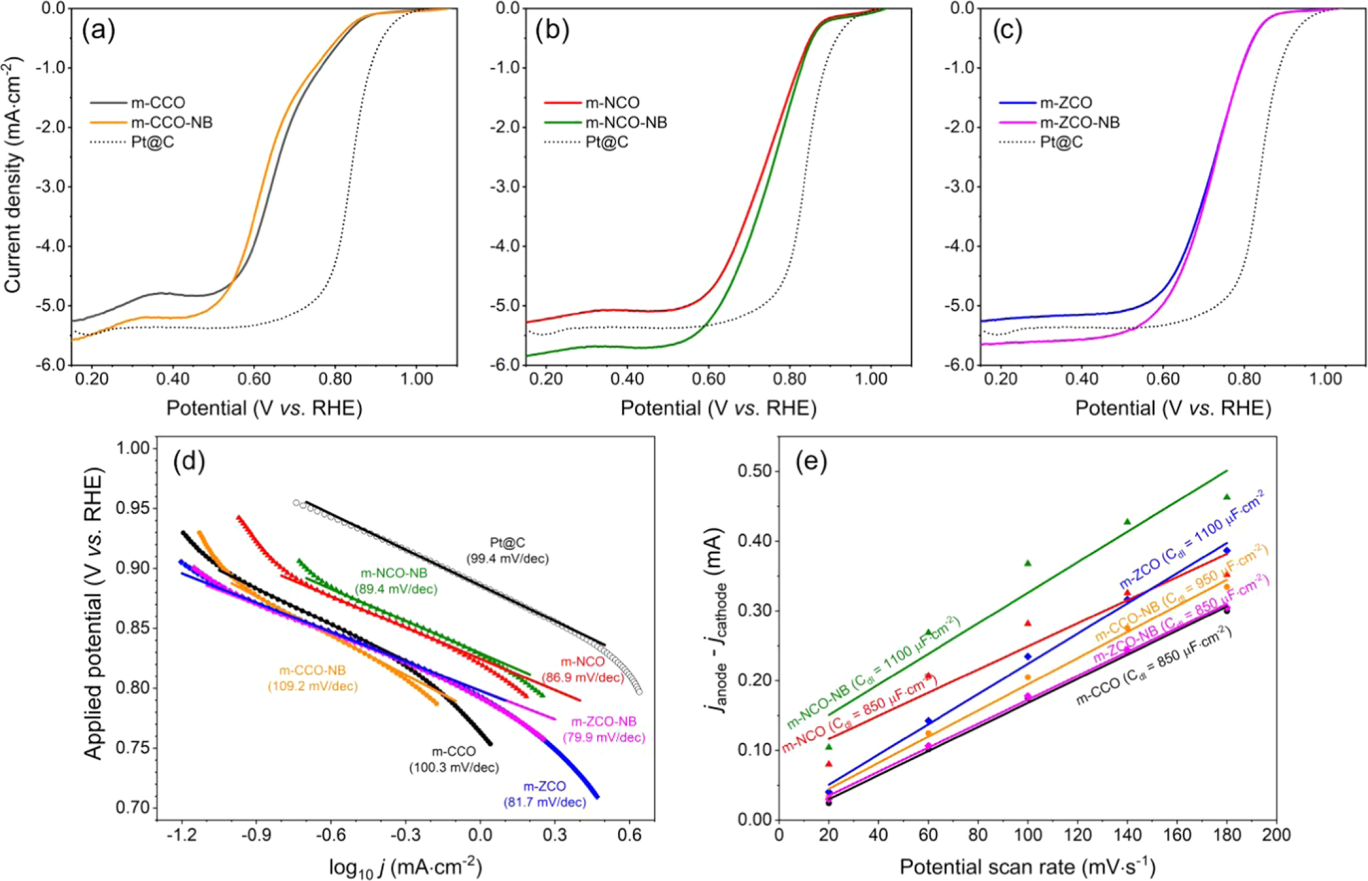
Comparative plot of ORR-LSV curves of (a) m-CCO and m-CCO-NB, (b) m-NCO and m-NCO-NB, and (c) m-ZCO and m-ZCO-NB samples in O_2_-saturated 0.1 M KOH solution at 25 °C and 1600 rpm. The LSV curve of platinum on graphitized carbon (Pt@C, dotted line) was introduced as a standard material for comparison. (d) Tafel slope and (e) electrochemical double-layered capacitance (*C*_dl_) of the prepared samples.

The catalytic activity of the prepared catalysts toward the ORR was evaluated by performing linear sweep voltammetry (LSV) in O_2_-saturated 0.1 M KOH solution at a scan rate of 10 mV·s^–1^ and a WE rotating speed of 1600 rpm. The comparison between the ORR-LSV curves of the pristine and post-NaBH_4_-treated oxides is shown in Figure 7a–c. Both m-NCO and m-ZCO catalysts showed better ORR catalytic activity than that of the m-CCO catalyst as viewed from the lower required electrode potential (*E*) to reach the benchmark current of −3.0 mA·cm^–2^ (Table S13).^3,7,68^ The results are in agreement with the Tafel slopes (Figure 7d) of the m-NCO (86.9 mV·dec^–1^) and m-ZCO (81.7 mV·dec^–1^), which are lower than those of the m-CCO (100.3 mV·dec^–1^) and Pt@C (99.4 mV·dec^–1^). These results confirm the improved kinetics of the mixed metal oxides as a possible result of strong electronic interactions between the metals, which would lead to the synergistic effect and provide more active sites for catalyzing the ORR. Furthermore, the improved kinetics might be caused by the increased defect concentration achieved upon the cation substitution. Here, the effect of NaBH_4_ treatment is of interest because all NaBH_4_-treated samples show enhanced catalytic performance (Table S13). The m-NCO-NB catalyst outperforms the other catalysts in terms of the electrode potential (0.720 V vs RHE at −3.0 mA·cm^–2^) and half-wave potential (*E*_1/2_) of 0.745 vs RHE (Table S13). Meanwhile, the m-ZCO-NB catalyst showed the lowest Tafel slope of 79.9 mV·dec^–1^ (Figure 7d and Table S13), indicating a catalyst with the most rapid kinetics among the samples. In addition to the activity tests, a stability test was performed for all the catalysts. The accelerated durability test (ADT) was carried out for 1000 CV cycles, as described in the experimental part. The results in Figure S13 proved the high stability of the prepared catalysts in the ORR.

Evaluation of catalyst *C*_dl_ and ECSA provides additional insights into the effect of the NaBH_4_ treatment, which leads to an increased ECSA value for m-CCO-NB and m-NCO-NB but a decreased value for m-ZCO-NB (Figures 7e and S14 and Table S13). Please note that the observed ECSA in the ORR in all cases is higher than that in the OER due to additional ECSA from the carbon black additive in the ORR ink. Through this, the carbon black that primarily catalyzed the ORR through the 2 e^–^ pathway would reduce the overall number of transferred electrons.^69^ Nevertheless, the observed number of electrons transferred (*n*) as approximated by the Koutecký–Levich analysis^2,51^ is close to 4 (Table S13). This suggests that the reaction is catalyzed mostly by the metal oxides rather than the carbon black support.

**Figure 8:**
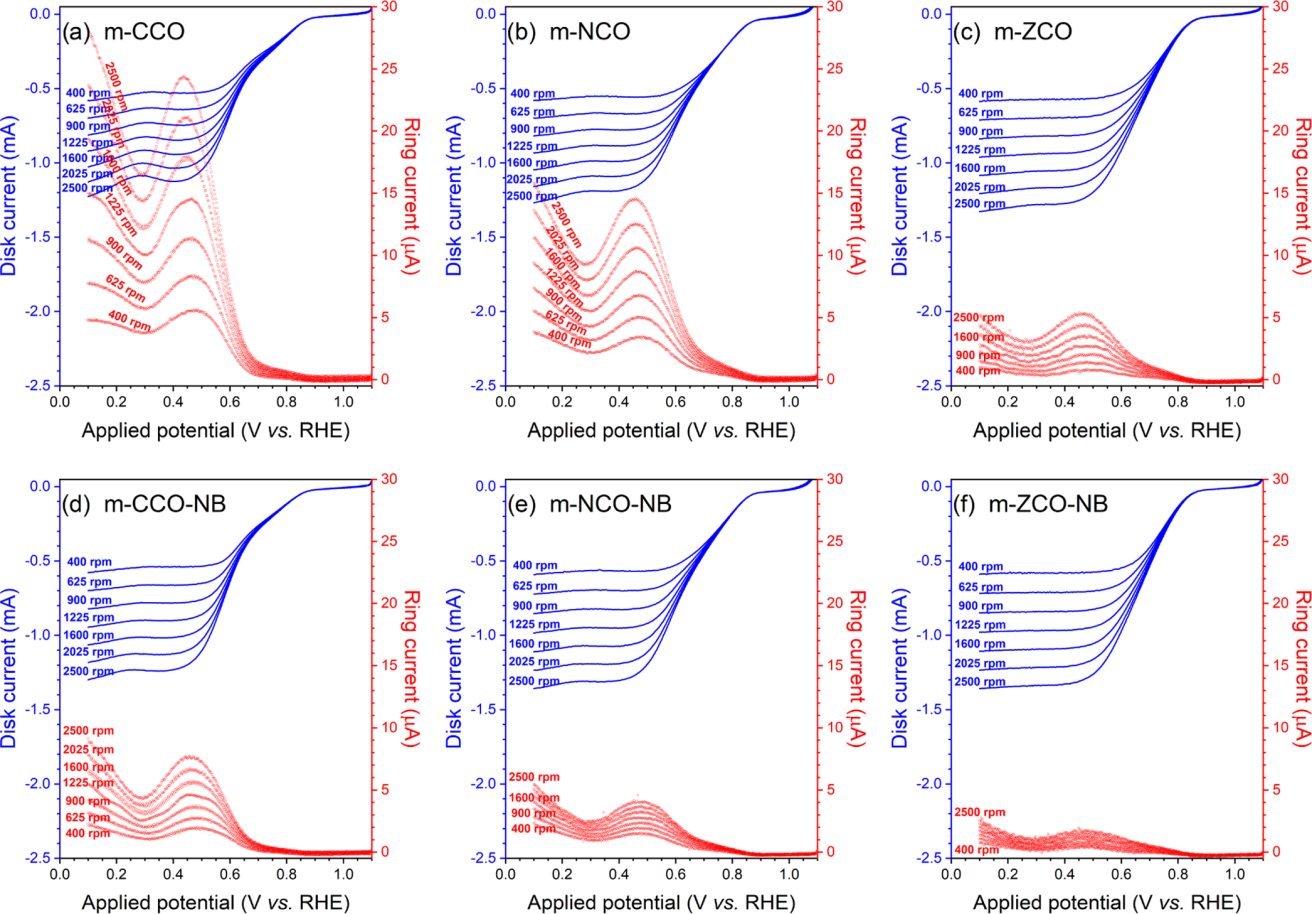
Responsive RRDE disk current (*I*_D_, blue) and ring current (*I*_R_, red) measured at different electrode rotating speeds in the ORR of (a) m-CCO, (b) m-NCO, (c) m-ZCO, (d) m-CCO-NB, (e) m-NCO-NB, and (f) m-ZCO-NB samples in O_2_-saturated 0.1 M KOH solution at 25 °C.

To provide more insights into the ORR kinetics, a detailed study of the RRDE experiments was carried out (Figure 8). To detect the side reaction product (HO_2_^–^), we repeated the experiments using the RRDE with the Pt ring.^2^ The potential applied on the Pt ring (1.476 V vs RHE) was sufficient to reoxidize these intermediates.^2^ Therefore, the higher the responsive current from the Pt ring (*I*_R_), the more facilitated the side reaction. As shown in Figure 8a–c, the disk current (*I*_D_) was observed along with the negative potential sweep until reaching the diffusion-controlled region (0.1 to ∼0.5 V vs RHE). In the meantime, the increasing *I*_R_ response during the potential sweep indicates the reoxidation of HO_2_^–^.^2^ By the Koutecký–Levich relationship, the higher the electrode rotating speed, the better the diffusion and the higher the current on the disk. The pristine catalysts produce a similar range of *I*_D_ responses at each given rotating speed. However, their corresponding *I*_R_ magnitude was different, underlining different tendencies of those catalysts to favor the side reaction (Figure 8a–c). The m-NCO and m-ZCO catalysts are less likely to lead to the side reaction than the m-CCO catalysts, which might be assigned to the influence of the enhanced surface defect concentration on ORR activity. The *I*_R_ and *I*_D_ responses of the m-CCO and m-NCO catalysts are different from those of the m-ZCO catalyst regarding the wave-formed signal, as observed in Figure 8. The presence of the wave-formed signal indicates a specific potential range, in which the 4 e^–^ pathway was more likely to occur, ca. 0.30–0.50 V vs RHE. For the potential lower than 0.30 V vs RHE, more concentration of HO_2_^–^ might be supplied by the ORR on carbon black.^69^ Therefore, the minimum *I*_R_ current in the m-ZCO sample indicates that this catalyst facilitates the 4 e^–^ pathway of the ORR the most among the pristine samples.

The influence of NaBH_4_ treatment can additionally be understood by comparing the *I*_R_ response of the pristine catalysts (Figure 8a–c) to their corresponding analogues (Figure 8d–f). A slight increase in the *I*_D_ response, associated with a dramatic decrease in the *I*_R_ response, was observed for all post-NaBH_4_-treated analogues. The best ORR catalyst in the present work, m-ZCO-NB, exhibits the most negligible *I*_R_ response. The number of electrons transferred (*n*) and the corresponding percentage of HO_2_^–^ production as a function of electrode potential, as represented in Figure S15, support this conclusion. The negligible percentage of the produced HO_2_^–^ (∼1%) in the diffusion-controlled region highlights the superiority of the m-ZCO-NB for ORR catalysis, and this sample shows the most favored 4 e^–^ pathway. Furthermore, information obtained from ECSA-normalized current densities, as shown in Figure S16a,c, reveals a slight drop in ORR activity of most catalytic sites after being treated with NaBH_4_. However, an exceptional case was found for the catalytic sites of m-ZCO-NB (Figure S16e), which exhibits the highest activity. On the other hand, the highest *S*_BET_-normalized current density was also obtained for m-ZCO-NB (Figure S16b,d,f). Therefore, these results underline the superior activity of the m-ZCO-NB material that outperformed the other catalysts in this study.

According to the results, zinc-substituted cobalt oxide has fulfilled the basic requirements of bifunctional catalysts, i.e., having high ORR/OER performance, being durable, less toxic, and more economical. Therefore, one can foresee such ordered mesoporous ZnCo_2_O_4_ to be a suitable material for renewable energy devices, as a growing number of reports utilize this type of compositions as catalysts in zinc–air batteries, regenerative fuel cells, and supercapacitor technologies. It is also interesting to envision ZnCo_2_O_4_ as a catalyst in other important reactions, i.e., hydrogen evolution reaction (HER). Overall, a better understanding of defect engineering in mixed metal spinel oxides will be helpful to advance renewable energy technologies, eventually to reach a sustainable clean energy society in the future.

## Conclusions

In this work, pure cobalt oxide and nickel- and zinc-substituted cobalt spinel oxides with similar mesoporous structures, specific surface areas (*S*_BET_ ≈ 110 cm^2^·g^–1^), and narrow pore size distribution were synthesized via a one-step impregnation technique, using KIT-6 silica as a template. In-depth material characterization by XPS and PALS suggests that incorporating nickel and zinc causes an increase in the oxygen vacancy concentration, which enhances the ORR and OER activity of the catalysts. High OER activity of Zn–Co oxide (m-ZCO) might be originated from the highly reactive catalytic sites and low charge transfer resistance (*R*_CT_) of 9.7 Ω, as a result of the strong electronic interactions between the metals and defect-rich structure of the catalyst. Moreover, this catalyst exhibits high activity in the ORR and promotes the 4 e^–^ pathway, which is beneficial for efficient ORR catalysis. In addition, for the first time, the role of surface post-treatment with NaBH_4_ was studied with the help of bulk characterization techniques, such as PALS. We suggest that this treatment modifies the surface, producing a high number of the metal-hydroxylated (M-OH) sites and more surface vacancy defects. For the Co oxide sample, although the treatment reduced the ECSA and slightly increased *R*_CT_, m-CCO appears to benefit from the surface modification via NaBH_4_ treatment, leading to a superior OER activity. Meanwhile, in the case of the mixed metal oxides, this post-treatment seems to be more suitable for improving ORR performances. Therefore, the superior ORR activity of the NaBH_4_-treated catalysts might be explained by the combination of a modified surface and a synergistic effect between Zn and Co ions. This study indicates the advantage of zinc substitution that not only improves the catalytic activity of the cobalt oxide spinel but also reduces the major drawback of the cobalt utilization. Therefore, Zn-substituted Co oxide is a promising bifunctional ORR/OER catalyst. Regarding the excellent ORR reactivity, one can foresee this type of catalysts to be interesting for a diversity of other applications related to oxygen electrocatalysis under alkaline conditions, e.g., metal–air batteries or regenerative fuel cells. However, the next and probably most important step would be to analyze the long-term stability and to ensure the durability of the materials. Online dissolution studies of the catalysts under electrochemical conditions, accelerated stress tests, and their further utilization in membrane electrode assembly setups (MEAs) or in aprotic Li-O_2_ cells^61^ are planned as future steps.

## Supporting Information Available

Physical characteristics of the KIT-6 silica template, Rietveld refinement fitting of the powder XRD patterns, SEM/EDX and ICP-MS elemental analysis results, theoretical model of bulk Co_3_O_4_ for positron lifetime simulation, simulated and observed positron lifetime from PALS, high-resolution XPS spectra of O 1s, Co 2p Ni 2p, and Zn 2p and deconvolutions, EDLC in the OER, cyclic voltammograms, Nyquist and Bode plots of material impedance in the OER, equivalent circuit for EIS fitting and results, chronopotentiometry profile of m-CCO-NB in the OER, ECSA- and *S*_BET_-normalized current density (OER), accelerated durability test (ADT) in the ORR, EDLC in the ORR, number of electrons transferred and HO_2_^–^ % yield calculated from disk and ring currents at different potentials in the ORR, and ECSA- and *S*_BET_-normalized current density (ORR) (PDF)
