# Supplementary material for: Defect-Engineered Hydroxylated Mesoporous Spinel Oxides as Bifunctional Electrocatalysts for Oxygen Reduction and Evolution Reactions
Source: ACS Appl Mater Interfaces. 2022 May 13;14(20):23307–21. doi: 10.1021/acsami.2c00254 (PMC9136850; doi:10.1021/acsami.2c00254)
Supplement: Supplementary file 1 — am2c00254_si_001.pdf [file am2c00254_si_001.pdf]

Supporting Information

Defect-engineered Hydroxylated Mesoporous Spinel Oxides as Bifunctional Electrocatalysts for Oxygen Reduction and Evolution Reactions

Wanchai Deeloed,[#†‡] *Tatiana Priamushko,*[#†] *Jakub Čížek,*[§] *Songwut Suramitr,*[‡] *and Freddy Kleitz**[†]

[†]Department of Inorganic Chemistry − Functional Materials, Faculty of Chemistry, University of Vienna, A-1090 Wien, Austria.

[‡]Department of Chemistry, Faculty of Science, Kasetsart University, Bangkok 10900, Thailand.

[§]Department of Low-Temperature Physics, Faculty of Mathematics and Physics, Charles University, V Holešovičkách 2, CZ-180 00 Praha 8, Czech Republic.

* Corresponding author: freddy.kleitz@univie.ac.at

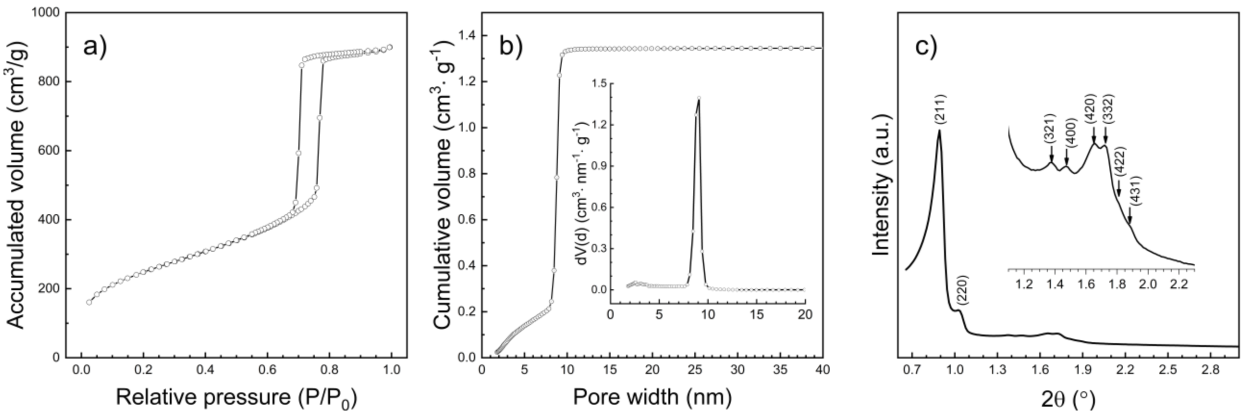

Figure S1. (a) N_2 physisorption isotherm at – 196 °C (77 K), (b) NLDFT pore size distribution, and (c) low-angle XRD pattern of the prepared KIT-6 (100 °C) silica template.

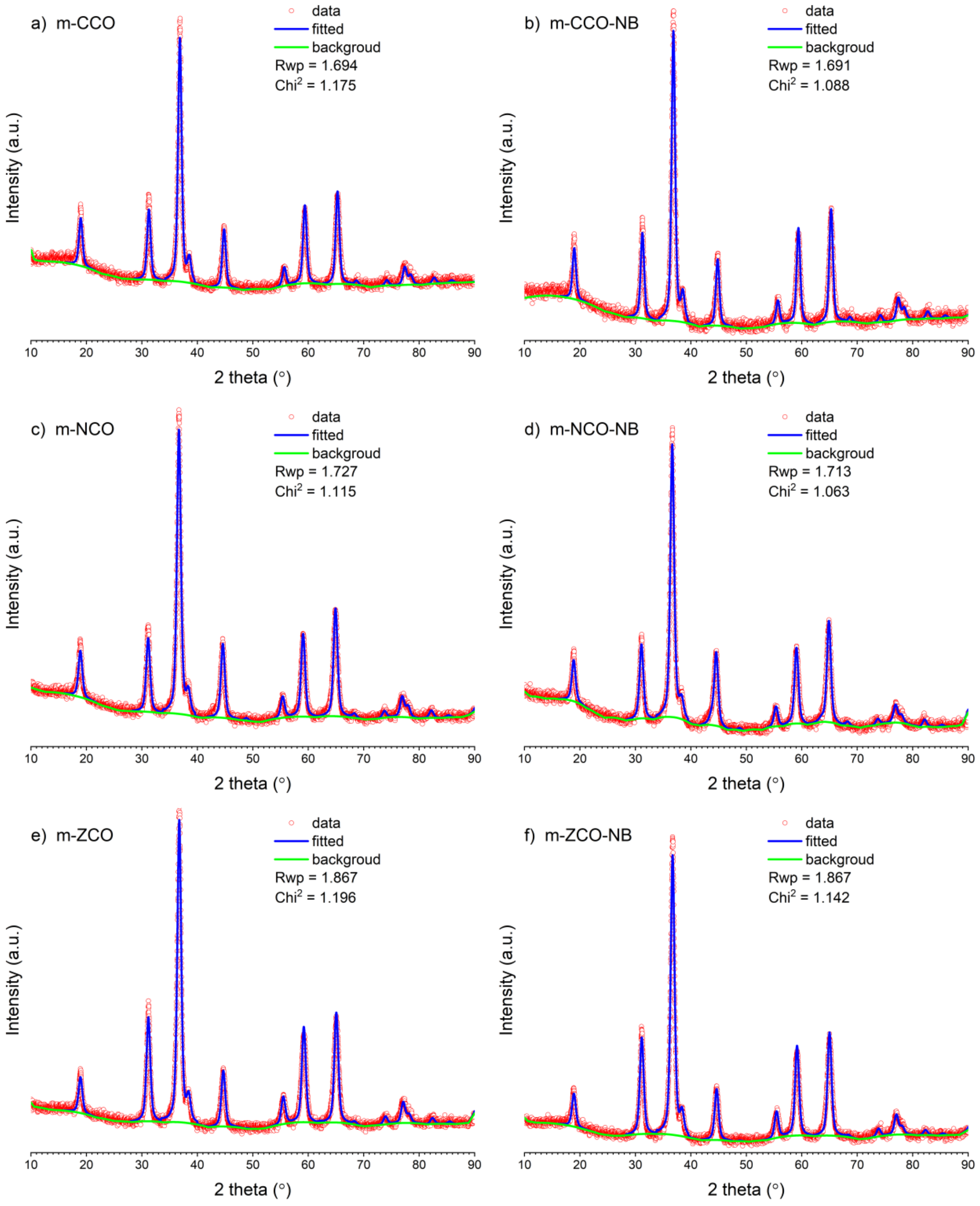

Figure S2. Rietveld refinement fitting of the obtained powder XRD patterns of (a) m-CCO, (b) m-CCO-NB, (c) m-NCO, (d) m-NCO-NB, (e) m-ZCO, and (f) m-ZCO-NB samples.

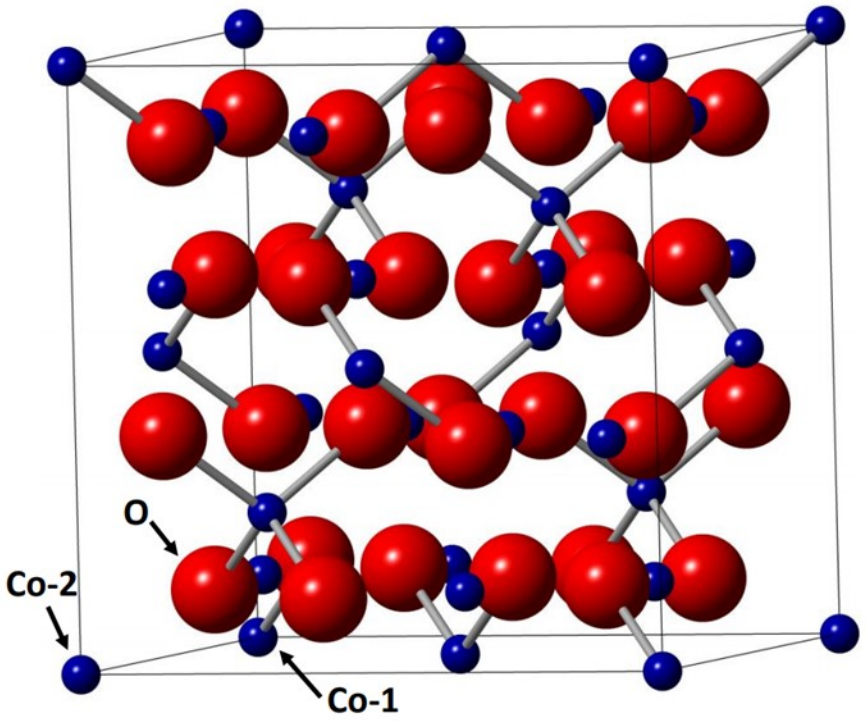

Figure S3. Model structure of bulk Co_3O_4 with the indicated position of the Co-1 (multiplicity 16), Co-2 (multiplicity 8), and oxygen ions. (Crystal Maker, version 2.2, http://crystalmaker.com/)

Calculations of positron lifetimes were performed using the density functional theory (DFT) within so-called standard scheme.[1] In this approximation, the positron density is assumed to be vanishingly small everywhere and to not affect the bulk electron structure. Positron density was calculated by numerical solution of the single-particle Schrödinger equation for positron in effective potential formed by ions and electrons. Electron density was constructed using atomic superposition.[1] The electron-positron correlation was treated within the generalized gradient approximation employing the approach introduced by Barbiellini *et al*.[2]

Lattice defects were modelled using 3584 atom-based supercells (4 × 4 × 4 unit cells with spinel structure and lattice parameter a = 8.0812 Å). Vacancies were created by removing corresponding atoms from the supercell.

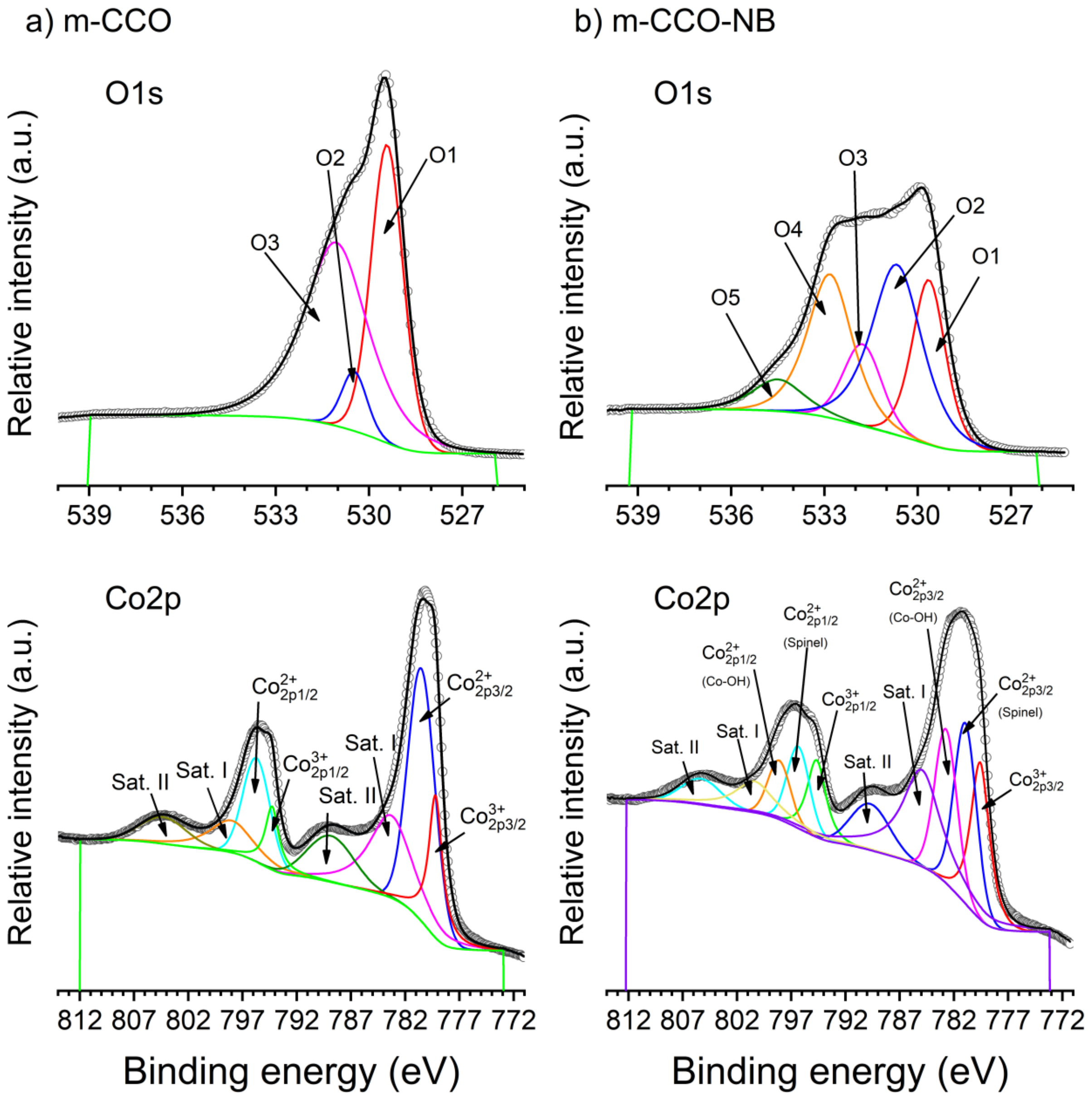

Figure S4. Deconvolution of O 1s and Co 2p XPS spectra from (a) m-CCO and (b) m-CCO-NB samples.

The large change in the Co 2p high-resolution spectra originates from the formation of Co-OH species on the surface of the m-CCO-NB sample, characterized by a Co^{2+} (Co-OH) peak (782.5 eV). Meanwhile, surface transformation also leads to a drastic change of O 1s spectrum. It was observed that the $NaBH_4$-treatment reduced the contribution of lattice oxygen (O1, 529.6 eV) but increased the hydroxyl oxygen in Co-OH (O2, 530.7 eV) confirming the existence of surface transformation. Finally, the newly formed Co-OH surface promoted the adsorption of oxygen (O4, 532.8 eV) and water molecules (O5, 534.5 eV) on the m-CCO-NB sample. These assumptions are also supported by the published literature.[10]

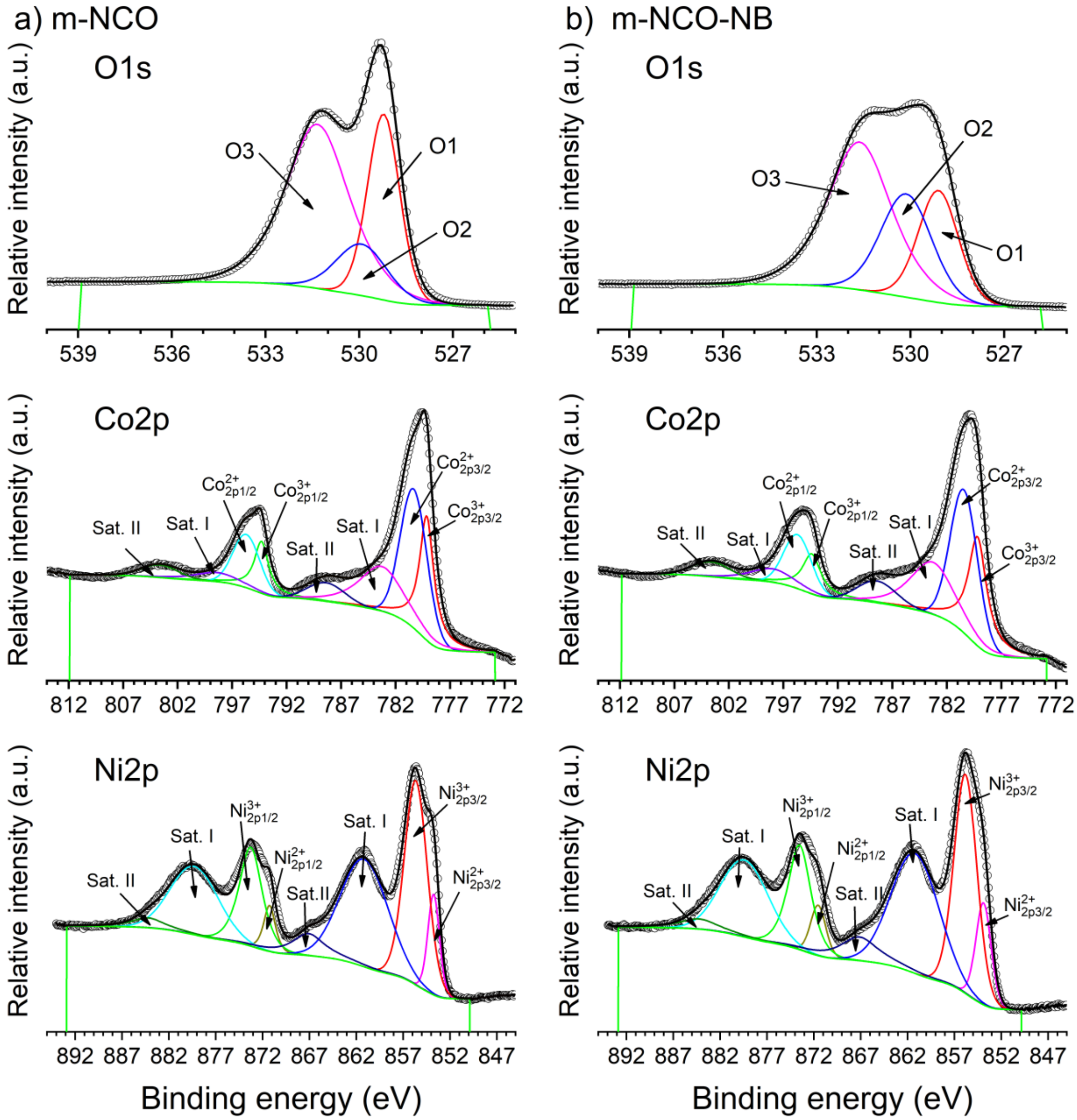

Figure S5. Deconvolution of O 1s, Co 2p, and Ni 2p XPS spectra from (a) m-NCO and (b) m-NCO-NB samples.

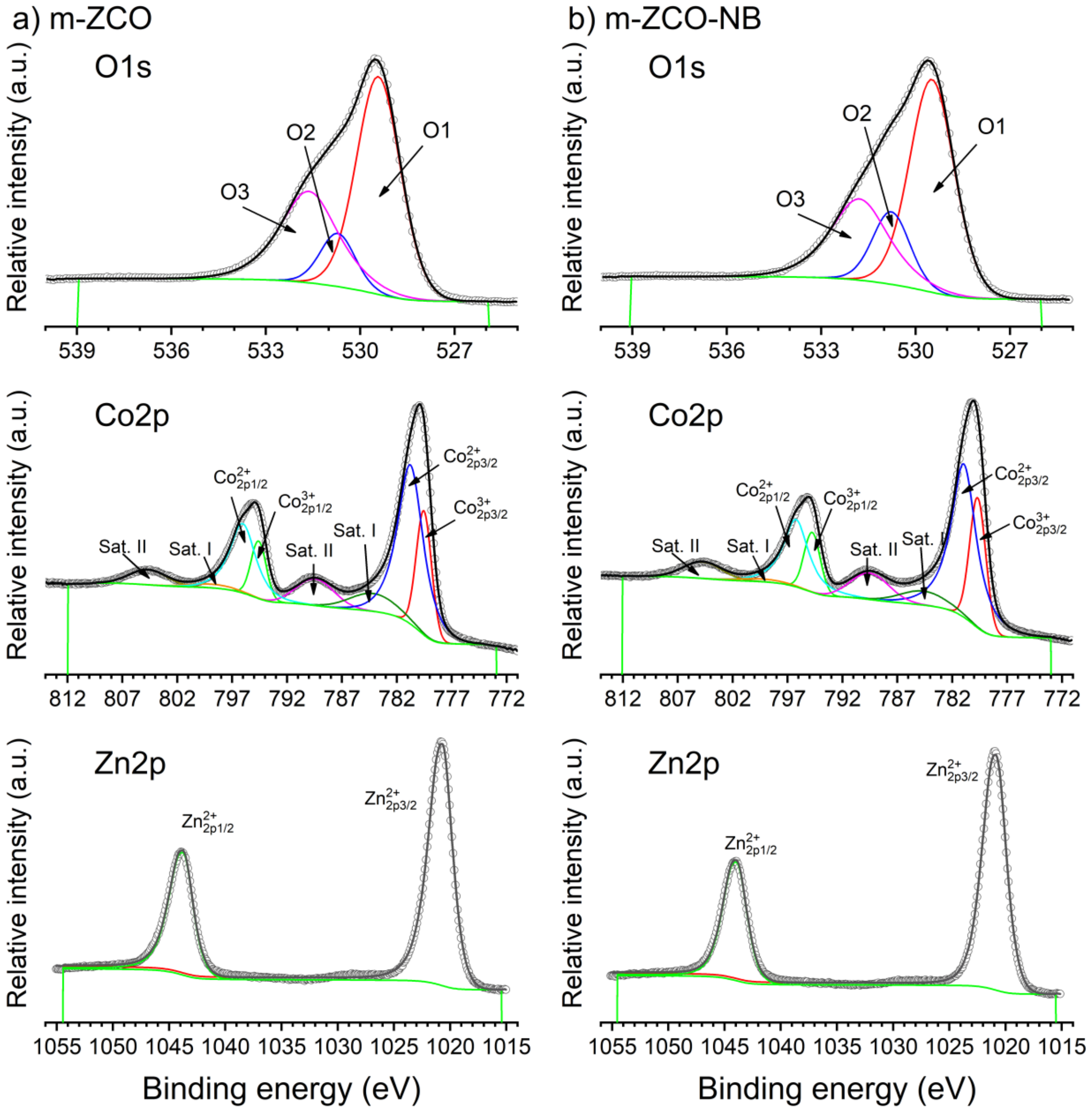

Figure S6. Deconvolution of O 1s, Co 2p, and Zn 2p XPS spectra from (a) m-ZCO and (b) m-ZCO-NB samples.

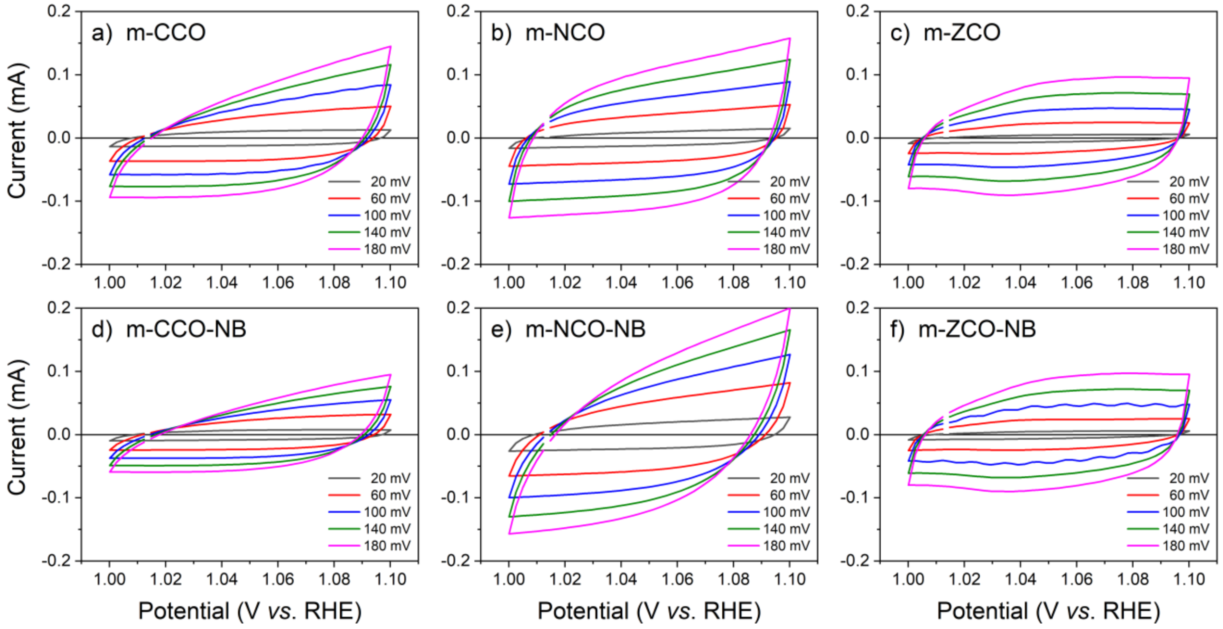

Figure S7. Electrochemical double-layered capacitance (EDLC) in OER for each catalyst (N_2-saturated 1.0 M KOH solution at 25 °C, RDE electrode rotating speed = 2000 rpm).

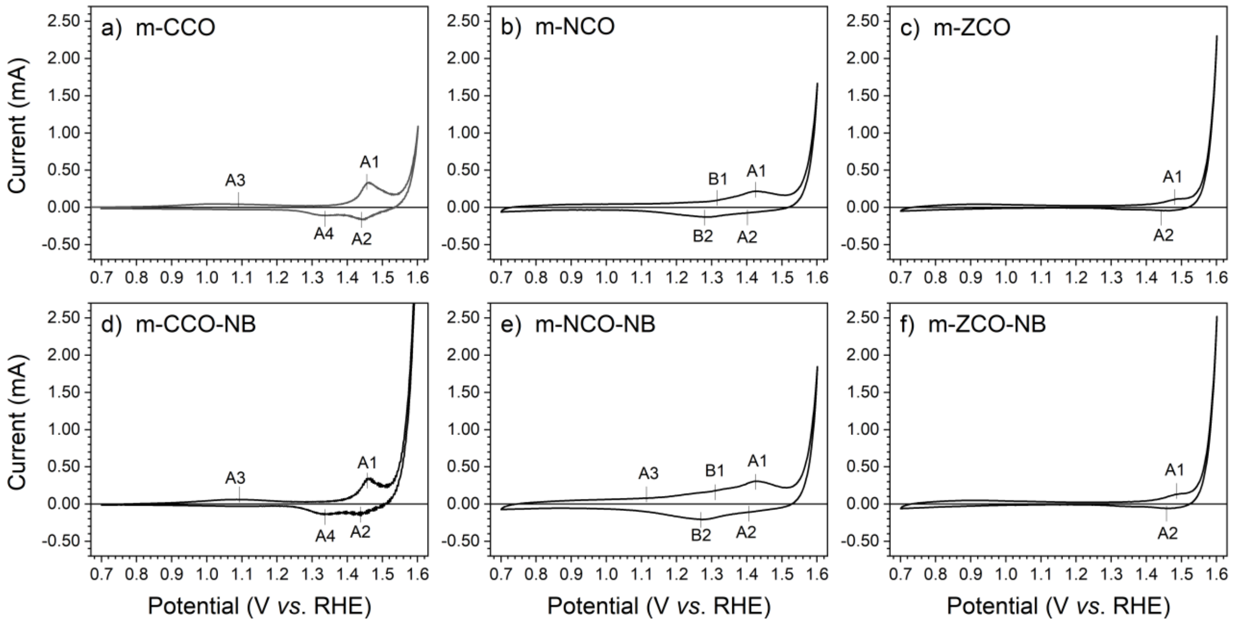

Figure S8. Cyclic voltammogram in OER for each catalyst (N_2-saturated 1.0 M KOH solution at 25 °C, RDE electrode rotating speed = 2000 rpm).

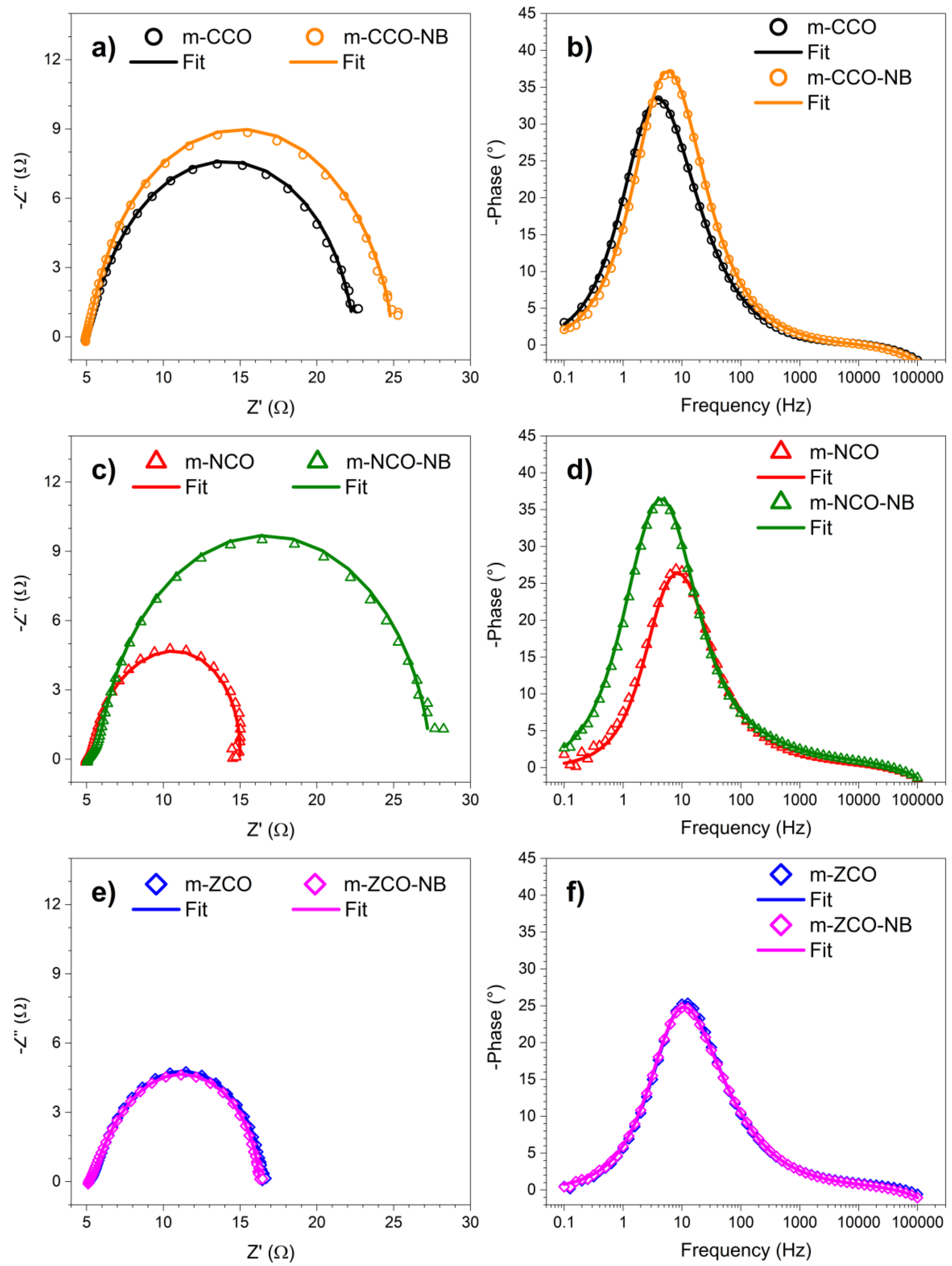

Figure S9. (a,c,e) Nyquist and (b,d,f) Bode plots for each catalyst in OER (N_2-saturated 1.0 M KOH solution at 25 °C, RDE electrode rotating speed = 2000 rpm).

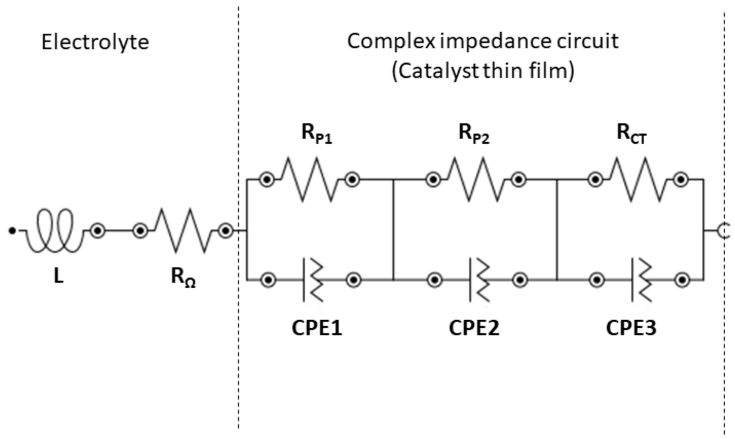

Figure S10. Equivalent circuit model for EIS spectra fitting.

The experimental impedances in Figure S9 were fitted to an equivalent circuit model concerning the presence of a passivation film, as proposed by Bredar *et al.*[3] The theoretical circuit model consists of two major parts, describing charge transport phenomena through the electrolyte and the catalyst mesoporous film. Ion-transport in electrolyte was described by the first circuit component, including an Inductant (L) and an Ohmic resistance (R_{Ω}). On the other hand, the second component, consisting of three RC circuit loops, imitates the complex impedance within the catalyst mesoporous film. The first (R_{P1}CPE1) and second (R_{P2}CPE2) loops have been introduced to explain the passivation layer and charge transport within the ordered mesopores sequentially. Besides, the third loop (R_{CT}CPE3) describes charge transport on the catalytic site, indicating an actual catalytic performance of catalyst.

The constant phase element (CPE) was used instead of the ideal capacitor, concerning the electrical double layer (EDL) that diverged from the theoretical capacitor behavior. The general impedance form of CPE can be written as $Z_{CPE} = [Y_0 (i\omega)^n]^{-1}$, where Y_0, *i, and* ω refer to the frequency-independent constant, square root of -1 and angular frequency of the ac signal, respectively.[3-5] The value of the n factor, ranging from -1 to 1, indicates the behavior of CPE in the circuit. For n = -1, 0, and 1, the CPE describes the pure inductor, pure resistor, and the ideal capacitor sequentially. Also, the CPE with n = 0.5 determines the intermediate behavior between resistor and capacitor (namely Warburg impedance, W).[4]

An excellent agreement between the theoretical and the experimental impedance was obtained in every case, indicating the reliable fitting parameters as shown in Table S12.

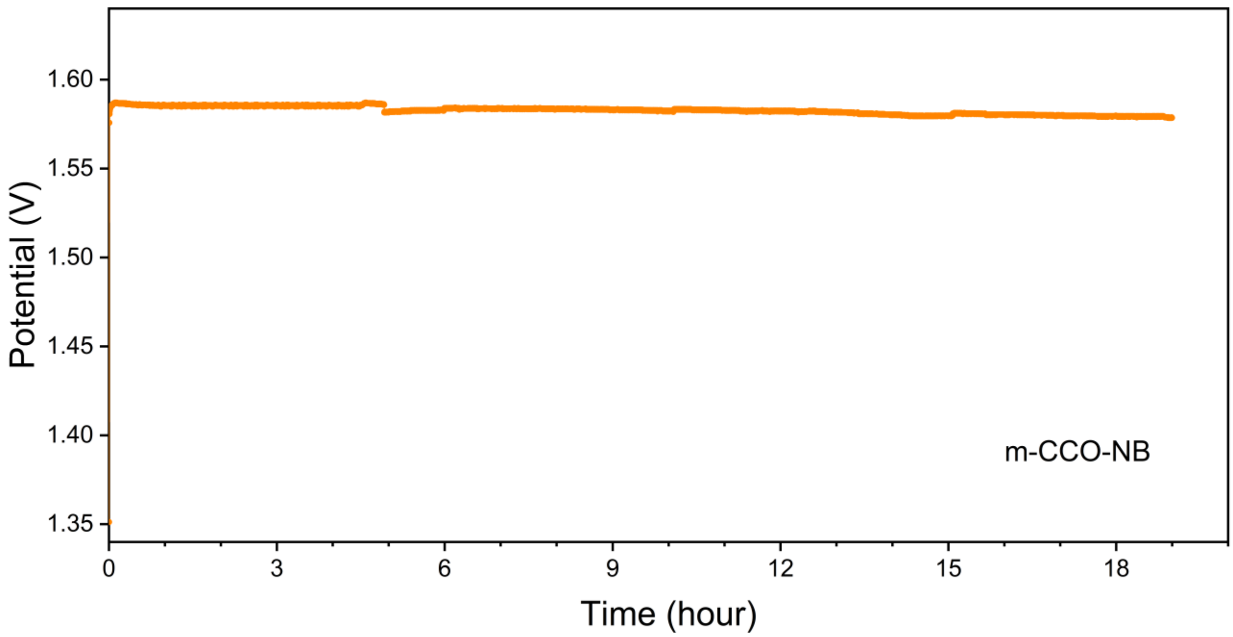

Figure S11. Chronopotentiometry profile of the m-CCO-NB sample (N_2-saturated 1.0 M KOH at room temperature, WE rotating speed = 2000 rpm, constant current density at 10 mA cm^{-2}).

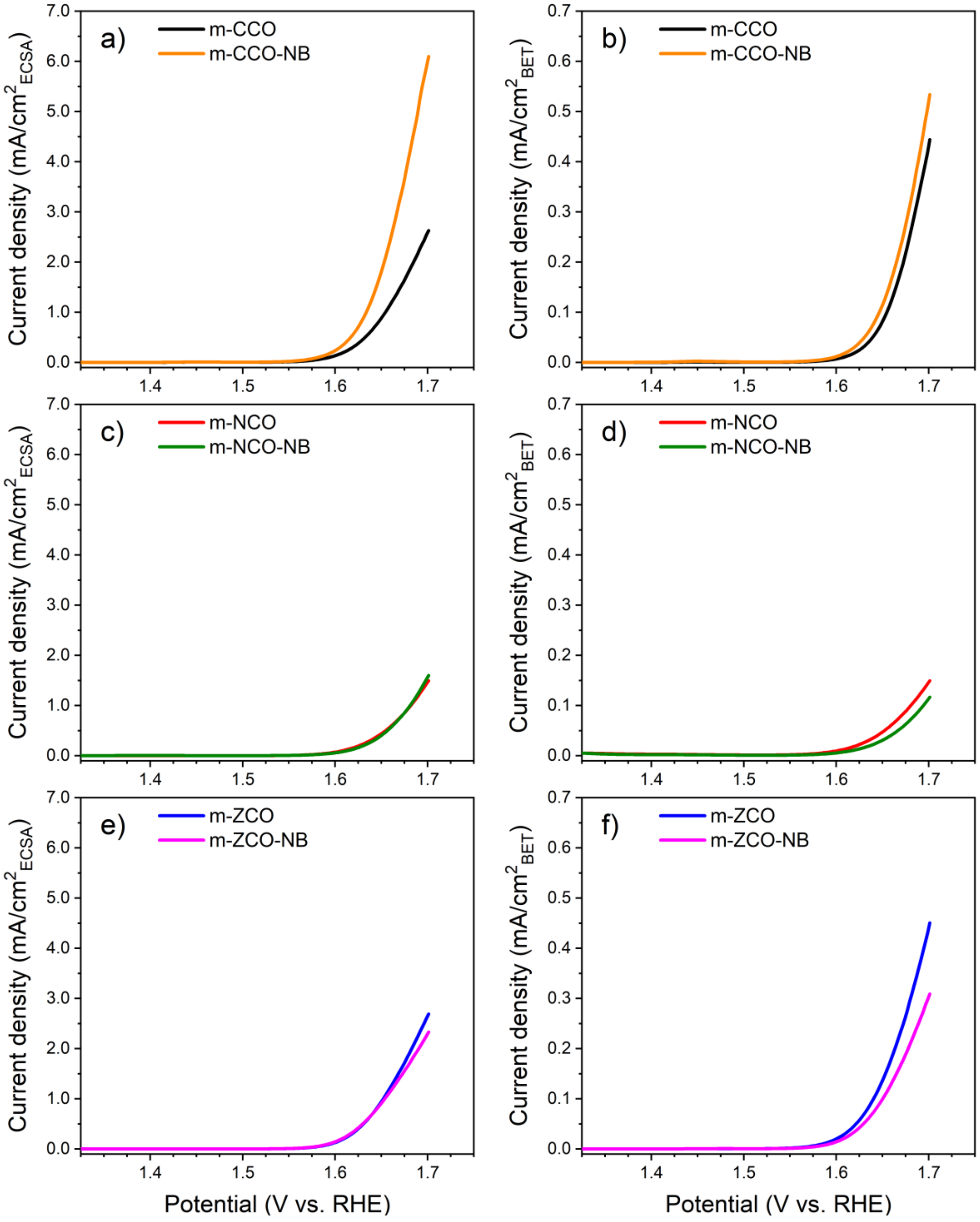

Figure S12. (a, c, e) ECSA-normalized current density and (b, d, f) S_{BET}-normalized current density of each catalyst in OER.

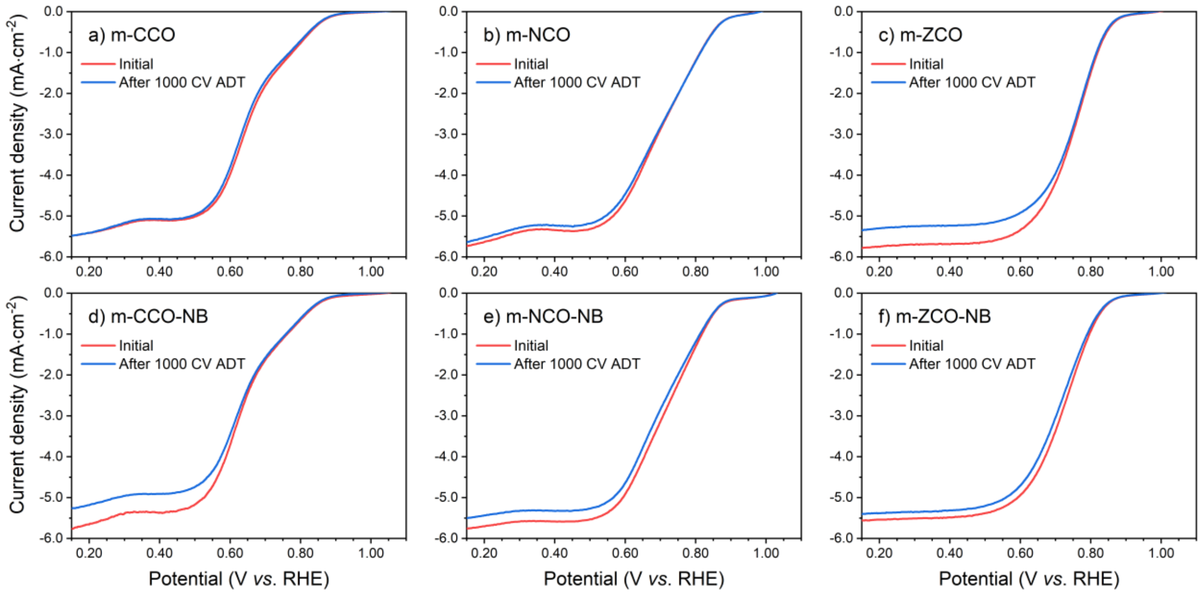

Figure S13. Accelerated-durability test (ADT) of the prepared materials in ORR (O_2-saturated 0.1 M KOH solution at 25 °C, RDE electrode rotating speed = 1600 rpm).

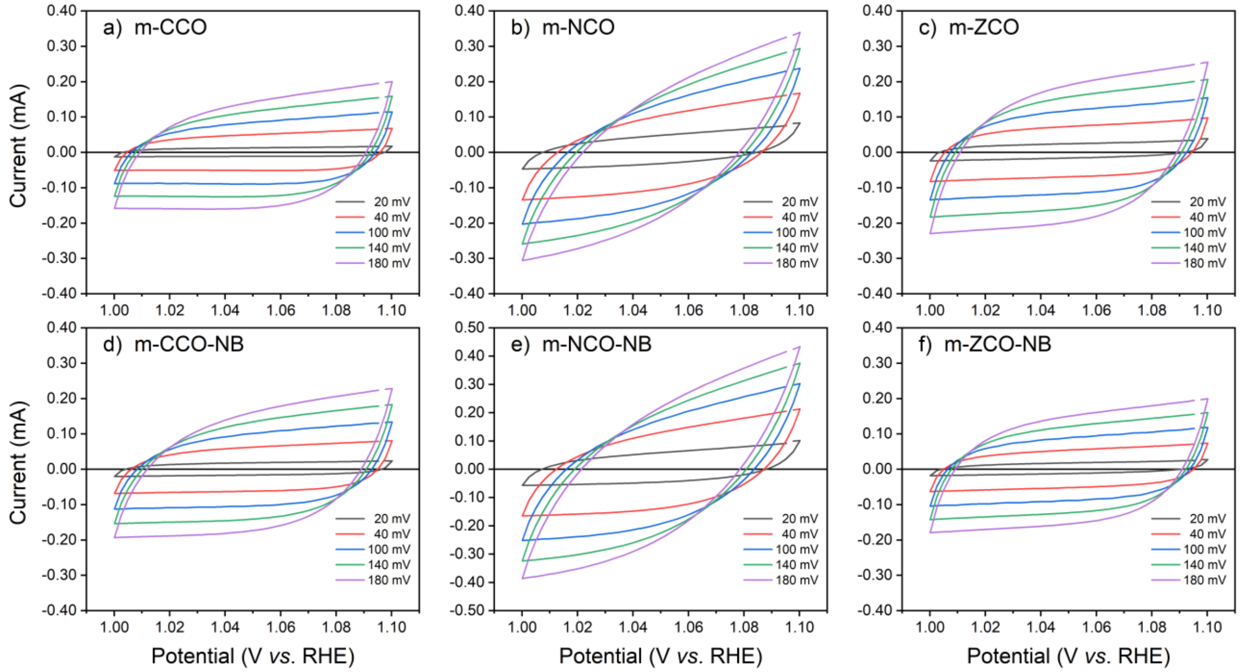

Figure S14. Electrochemical double-layered capacitance (EDLC) at various scan rates of the prepared materials in ORR after surface activation (O_2-saturated 0.1 M KOH solution at 25 °C, RDE electrode rotating speed = 1600 rpm).

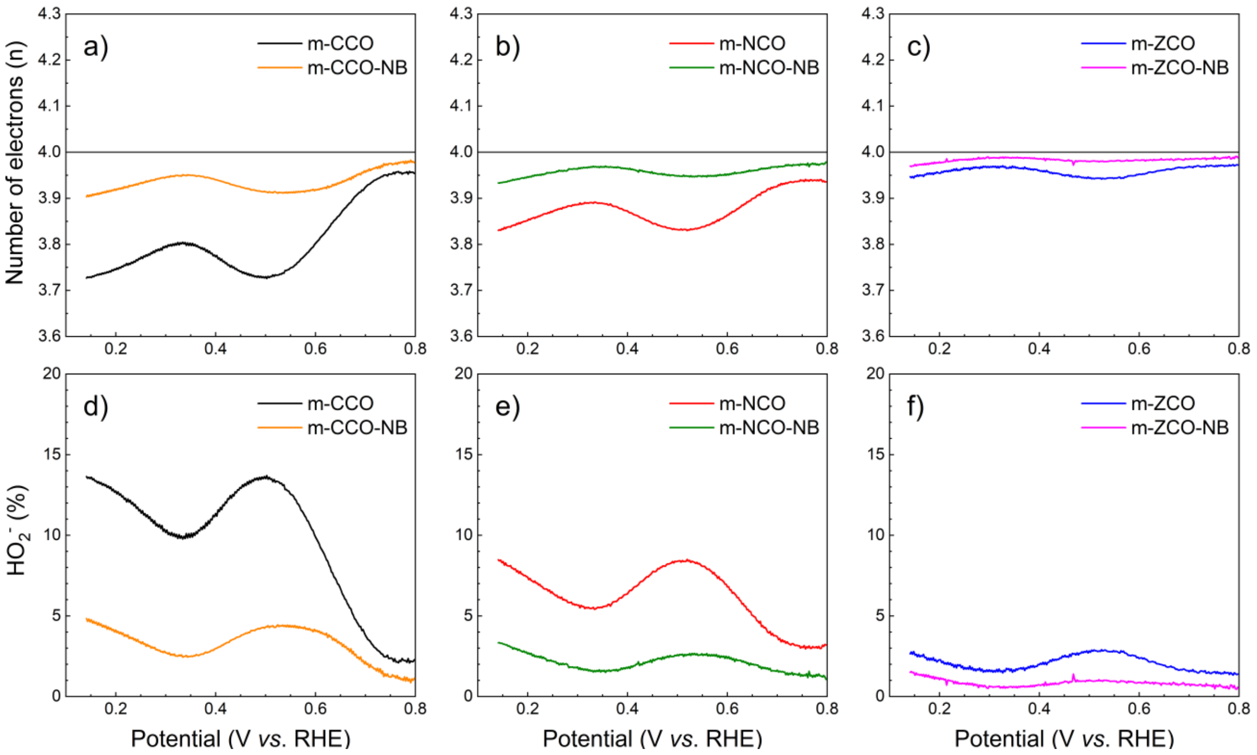

Figure S15. The number of electrons transferred (*n*) and percentage of HO_2^- produced in ORR of (a, d) m-CCO and m-CCO-NB, (b, e) m-NCO and m-NCO-NB and (c, f) m-ZCO and m-ZCO-NB samples. The calculated value was plotted against the swept potential on the RRDE electrode in N_2-saturated and O_2-saturated 0.1 M KOH solutions at 25 °C (RRDE electrode rotating speed = 1600 rpm, Pt ring potential = 1.476 V *vs.* RHE.

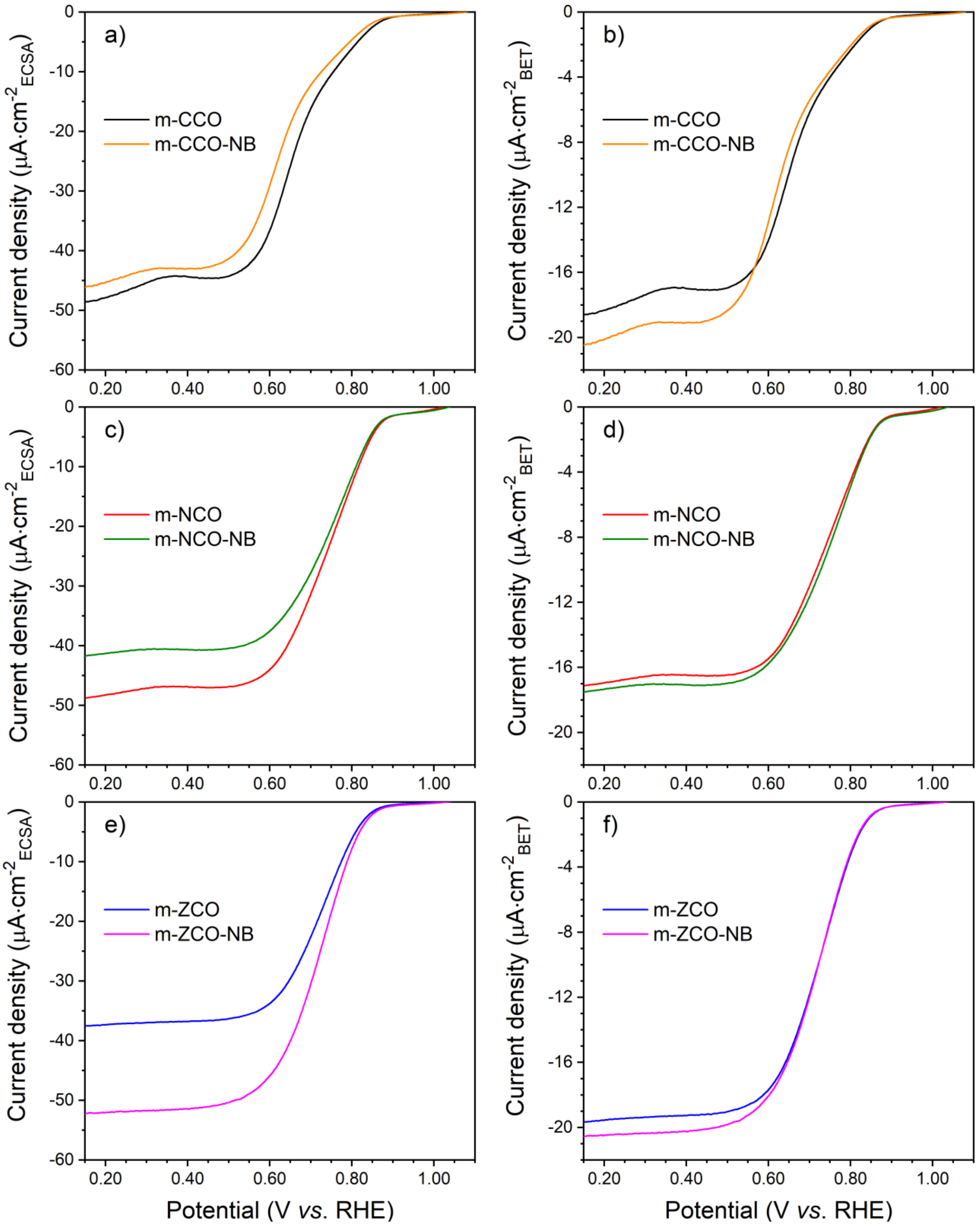

Figure S16. (a, c, e) ECSA-normalized current density and (b, d, f) S_{BET}-normalized current density of each catalyst in ORR.

Table S1. Refined lattice parameters of the synthesized samples.

Sample	Phase	wt.%	Space Group	a = b = c (Å)	α = β = γ (°)	V (Å^3)	Crystallite size (Å)	Reference pattern
m-CCO	$CoCo_2O_4$	100	Fd-3m	8.0812	90.0	527.76	105	PDF 98-006-9365
m-CCO-NB	$CoCo_2O_4$	100	Fd-3m	8.0810	90.0	527.71	107	PDF 98-006-9365
m-NCO	$NiCo_2O_4$	93	Fd-3m	8.1248	90.0	536.36	87	PDF 98-002-4211
	NiO	7	Fm-3m	4.1370	90.0	70.80	41	PDF 98-006-1318
m-NCO-NB	$NiCo_2O_4$	94	Fd-3m	8.1249	90.0	536.36	91	PDF 98-002-4211
	NiO	6	Fm-3m	4.1370	90.0	70.80	83	PDF 98-006-1318
m-ZCO	$ZnCo_2O_4$	100	Fd-3m	8.1067	90.0	532.77	89	PDF 98-007-3756
m-ZCO-NB	$ZnCo_2O_4$	100	Fd-3m	8.1126	90.0	533.94	91	PDF 98-007-3756

Table S2. Refined atomic coordinates and site occupancy of each synthesized sample.

Sample	Phase	Atom	x	y	z	Occ.	Site	Sym.
m-CCO	Co_3O_4	O1	0.23730	0.23730	0.23730	1.000	32e	.3m
		Co1	0.00000	0.00000	0.00000	0.984	16c	.-3m
		Co2	0.37500	0.37500	0.37500	0.912	8b	-43m
m-CCO-NB	Co_3O_4	O1	0.23730	0.23730	0.23730	1.000	32e	.3m
		Co1	0.00000	0.00000	0.00000	0.997	16c	.-3m
		Co2	0.37500	0.37500	0.37500	0.946	8b	-43m
m-NCO	$NiCo_2O_4$	O1	0.24300	0.24300	0.24300	1.000	32e	.3m
		Ni1	0.00000	0.00000	0.00000	0.416	16c	.-3m
		Co1	0.00000	0.00000	0.00000	0.484	16c	.-3m
		Co2	0.37500	0.37500	0.37500	0.814	8b	-43m
	NiO	O1	0.50000	0.50000	0.50000	0.941	4b	m-3m
		Ni1	0.00000	0.00000	0.00000	0.417	4a	m-3m
m-NCO-NB	$NiCo_2O_4$	O1	0.24300	0.24300	0.24300	1.000	32e	.3m
		Ni1	0.00000	0.00000	0.00000	0.443	16c	.-3m
		Co1	0.00000	0.00000	0.00000	0.438	16c	.-3m
		Co2	0.37500	0.37500	0.37500	0.828	8b	-43m
	NiO	O1	0.50000	0.50000	0.50000	1.000	4b	m-3m
		Ni1	0.00000	0.00000	0.00000	0.949	4a	m-3m
m-ZCO	$ZnCo_2O_4$	O1	0.23670	0.23670	0.23670	1.000	32e	.3m
		Co1	0.00000	0.00000	0.00000	0.955	16c	.-3m
		Zn1	0.00000	0.00000	0.00000	0.058	16c	.-3m
		Co2	0.37500	0.37500	0.37500	0.202	8b	-43m
		Co3	0.37500	0.37500	0.37500	0.102	8b	-43m
		Zn2	0.37500	0.37500	0.37500	0.671	8b	-43m
m-ZCO-NB	$ZnCo_2O_4$	O1	0.23670	0.23670	0.23670	1.000	32e	.3m
		Co1	0.00000	0.00000	0.00000	0.949	16c	.-3m
		Zn1	0.00000	0.00000	0.00000	0.057	16c	.-3m
		Co2	0.37500	0.37500	0.37500	0.194	8b	-43m
		Co3	0.37500	0.37500	0.37500	0.103	8b	-43m
		Zn2	0.37500	0.37500	0.37500	0.673	8b	-43m

Table S3. The elemental composition of the different samples, as obtained by spot-EDX.

Sample	Elemental composition (Atomic %)							n	Atomic ratio	
	O	B	Na	Si	Co	Ni	Zn		Ni:Co	Zn:Co
m-CCO<br>m-CCO-NB	62.98 ± 0.45	-	0.75 ± 0.10	0.66 ± 0.03	35.61 ± 0.47	-	-	10	-	-
m-NCO<br>m-NCO-NB	58.04 ± 8.30	-	-	1.65 ± .020	27.55 ± 6.35	12.75 ± 2.45	-	8	0.46	-
m-ZCO<br>m-ZCO-NB	58.75 ± 1.88	-	-	0.95 ± 0.09	28.48 ± 1.23	-	11.83 ± 1.30	9	-	0.42

Table S4. The elemental composition of the different samples, as obtained by area-EDX.

Sample	Elemental composition (Atomic %)							n	Atomic ratio	
	O	B	Na	Si	Co	Ni	Zn		Ni:Co	Zn:Co
m-CCO<br>m-CCO-NB	63.23 ± 0.06		0.71 ± 0.12	0.63 ± 0.04	35.42 ± 0.13	-	-	3	-	-
m-NCO<br>m-NCO-NB	62.32 ± 0.12	-	-	1.66 ± 0.03	24.30 ± 0.05	11.71 ± 0.05	-	4	0.48	-
m-ZCO<br>m-ZCO-NB	60.96 ± 2.05	-	-	0.88 ± 0.01	26.77 ± 1.54	-	11.39 ± 0.53	5	-	0.43

Table S5. The elemental composition of the different samples, as obtained by ICP-MS.

Sample	Elemental composition (Atomic %)							n	Atomic ratio	
	O	B	Na	Si	Co	Ni	Zn		Ni:Co	Zn:Co
m-CCO		-	-	<LOQ	70.79	-	-		-	-
m-CCO-NB		<LOQ	0.65	1.01	66.98	-	-		-	-
m-NCO		-	-	0.91	46.02	23.04	-		0.50	-
m-NCO-NB		0.28	0.61	0.92	44.42	22.18	-		0.50	-
m-ZCO		-	-	<LOQ	49.70	-	22.88		-	0.41
m-ZCO-NB	incomplete sample dissolution									

LOQ = limit of quantification; LOQ of B = 5.041 and Si = 20.908 $\mu g \cdot L^{-1}$.

Table S6. Theoretical positron's lifetimes (τ) and corresponding positron binding energies (E_B) in a perfect Co_3O_4 lattice (bulk) and various defects.

Positron state	τ (ps)	E_B (eV)
Bulk	145.5	
V_O	147.8	0.007
V_{Co-1}	174.8	0.495
V_{Co-2}	217.6	1.258
$V_{Co-1} + V_O$	200.3	0.995
$V_{Co-2} + V_O$	249.7	1.754

Table S6 shows calculated lifetimes for positron delocalized in a perfect Co_3O_4 lattice (bulk) and trapped at various defects in the model structure as shown in Figure S3. Positron delocalized in a perfect Co_3O_4 lattice exhibits a lifetime of τ_B = 145.5 ps. Oxygen vacancy (V_O) is only a shallow trap unable to confine the positron wave function. In Table S6, V_O is characterized by positron lifetime approaching τ_B and the binding energy is almost zero. There are two types of Co vacancy sites: Co-1 with multiplicity 16 and Co-2 with multiplicity 8, as indicated in Figure S3. Both types of Co vacancies (V_{Co}) are deep positron traps characterized by the lifetime of 174.8 and 217.6 ps for V_{Co-1} and V_{Co-2}, respectively. Calculated lifetimes for positrons trapped in V_{Co} + V_O di-vacancies state are listed in the table as well.

Table S7. Lifetimes (τ_i) and relative intensities (I_i) of the components resolved in positron lifetime spectra for each mesoporous oxide sample. Experimental uncertainties (one standard deviation) are shown in parentheses. The values $\Delta\tau_i$ indicate changes of the corresponding positron lifetime for $NaBH_4$ treated sample with respect to its value for non-treated sample.

Sample	τ_1 (ps)	$\Delta \tau_1$	I_1 (%)	τ_2 (ps)	$\Delta \tau_2$	I_2 (%)
m-CCO	239(5)		44(3)	387(4)		56(2)
m-CCO-NB	242(3)	+3	52(1)	455(4)	+68	48(1)
m-NCO	283(2)		68(2)	480(1)		32(2)
m-NCO-NB	296(2)	+13	73(2)	520(1)	+40	27(2)
m-ZCO	253(2)		67(1)	499(5)		33(1)
m-ZCO-NB	285(3)	+32	72(2)	512(8)	+13	28(2)

Table S8. Parameters of the deconvoluted contributions from the XPS spectra of m-CCO and m-CCO-NB samples.

Chemical species	m-CCO			m-CCO-NB			References
	B.E. (eV)	Peak area	Peak fraction	B.E. (eV)	Peak area	Peak fraction	
**O 1s**							
O1 (M-O-M)	529.4	84895.19	0.40	529.6	50161.29	0.21	[6-11]
O2 (M-OH)	530.5	13479.21	0.06	530.7	82220.93	0.35	[6-11]
O3 (Vacancies)	531.1	114857.56	0.54	531.8	27706.43	0.12	[6-11]
O4 (Chemisorb O)	-	-	-	532.8	60900.43	0.26	[9-12]
O5 (H_2O vapor.)	-	-	-	534.5	15372.35	0.07	[12]
**Co 2p**							
Co^{3+} (Spinel)	779.2	88189.36	0.28	779.6	111660.48	0.35	[6, 9-11, 13, 14]
Co^{2+} (Spinel)	780.5	228966.97	0.72	781.0	136185.71	0.42	[6, 9-11, 13, 14]
Co^{2+} (Co-OH)	-	-	-	782.5	72925.77	0.23	[11]

Table S9. Parameters of the deconvoluted contributions from the XPS spectra of m-NCO and m-NCO-NB samples.

Chemical species	m-NCO			m-NCO-NB			References
	B.E. (eV)	Peak area	Peak fraction	B.E. (eV)	Peak area	Peak fraction	
**O 1s**							
O1 (M-O-M)	529.2	64506.1	0.46	529.1	63513.95	0.31	[6-8]
O2 (M-OH)	529.9	17844.05	0.13	530.1	74726.93	0.28	[6-8]
O3 (Vacancies)	531.3	56959.81	0.41	531.6	141562.09	0.41	[6-8]
**Co 2p3/2**							
Co^{3+} (Spinel)	779.2	34059.57	0.47	779.2	77547.61	0.41	[6, 13, 14]
Co^{2+} (Spinel)	780.4	38070.85	0.53	780.4	109716.83	0.59	[6, 13, 14]
**Ni 2p3/2**							
Ni^{2+} (Spinel)	853.7	21445.22	0.33	853.9	38823.51	0.24	[6, 13, 14]
Ni^{3+} (Spinel)	855.6	43526.07	0.67	855.8	125041.95	0.76	[6, 13, 14]

Table S10. Parameters of the deconvoluted contributions from the XPS spectra of m-ZCO and m-ZCO-NB samples.

Chemical species	m-ZCO			m-ZCO-NB			References
	B.E. (eV)	Peak area	Peak fraction	B.E. (eV)	Peak area	Peak fraction	
**O 1s**							
O1 (M-O-M)	529.4	95524.14	0.56	529.5	100518.85	0.55	[6-8]
O2 (M-OH)	530.7	18527.36	0.11	530.8	28468.98	0.16	[6-8]
O3 (Vacancies)	531.6	56304.21	0.33	531.8	53178.15	0.29	[6-8]
**Co 2p3/2**							
Co^{3+} (Spinel)	779.5	59775.37	0.29	779.7	73470.92	0.31	[6, 13, 14]
Co^{2+} (Spinel)	780.8	146960.10	0.71	781.0	164892.26	0.69	[6, 13, 14]
**Zn 2p3/2**							
Zn^{2+} (Spinel)	1020.7	213735.23	1.00	1020.9	224747.54	1.00	[15]

Table S11. Electrochemical characteristics of the prepared samples and other relevant catalysts, measured on GC RDE electrode, in the OER experiments.

Sample	S_{BET} ($m^2 \cdot g^{-1}$)	Electrolyte	Catalyst loading ($mg \cdot cm^{-2}$)	j [a] ($mA \cdot cm^{-2}$)	Tafel slope ($mV \cdot dec^{-1}$)	η [b] (mV)	C_{dl} (μF)	ECSA (cm^2)	Reference
m-CCO	111	1 M KOH	0.25	144	53	378	430.10	10.75	This work
m-CCO-NB	107	1 M KOH	0.25	208	51	376	268.12	6.70	This work
m-NCO	121	1 M KOH	0.25	151	51	378	793.12	19.83	This work
m-NCO-NB	131	1 M KOH	0.25	119	56	390	584.20	14.60	This work
m-ZCO	105	1 M KOH	0.25	176	44	373	513.99	12.85	This work
m-ZCO-NB	108	1 M KOH	0.25	152	45	371	512.85	12.82	This work
Commercial samples:									
Co_3O_4	1.95	1 M NaOH	0.8	N/A	61	500	N/A	1.52	[16]
$NiCoO_2$	9.78	1 M NaOH	0.8	N/A	53	390	N/A	3.92	[16]
IrO_2	39.02	1 M NaOH	0.8	N/A	47.7	380	N/A	423.8	[16]
IrO_2	N/A	1 M KOH	1.02	N/A	121.8	341	N/A	N/A	[17]
RuO_2	11.38	1 M NaOH	0.8	N/A	64.6	380	N/A	45.52	[16]
Mixed-metal oxides:									
$NiCo_2O_4$	N/A	1 M KOH	NA	N/A	95	380	173.18	N/A	[18]
OM Co_3O_4 [c]	106	1 M KOH	0.12	155	50	379	N/A	N/A	[19]
OM $Ni_xCo_{(3-x)}O_4$ [c]	119	1 M KOH	0.12	140	54	373	750	19	[19]
OM $Cu_{0.2}Ni_{0.8}Co_2O_4$ [c]	107	1M KOH	0.12	198	37	312	820	21	[19]
OM $Cu_{0.5}Ni_{0.5}Co_2O_4$ [c]	118	1M KOH	0.12	165	37	334	N/A	N/A	[19]
$ZnCo_2O_4$	N/A	1 M KOH	1.02	N/A	207.5	408	N/A	16.17	[17]
$ZnCo_2O_4$ spindle	66	1 M KOH	0.12	N/A	59.5	389	N/A	N/A	[20]
$Zn_{0.4}Ni_{0.6}Co_2O_4$/NCNTs	123.2	0.1M KOH	0.071	N/A	118.3	412	N/A	N/A	[21]
Flower-like $NiCo_2O_4$	55.3	1M NaOH	1.0	N/A	109.88	350	N/A	N/A	[22]

[a] Current density (j) at the applied potential of 1.7 V *vs*. RHE.

[b] Overpotential (η) corresponding to the current density at 10 $mA \cdot cm^{-2}$.

[c] Ordered mesoporous oxides synthesized via nanocasting of KIT-6 (100 °C) silica template.

Table S12. Fitting parameters from EIS spectra of the prepared catalysts in the OER experiments.

Sample	L ($\times 10^{-9}$ H)	R_{Ω} (Ω)	R_{P1} (Ω)	CPE_1		R_{P2} (Ω)	CPE_2		R_{CT} (Ω)	CPE_3		χ^2 ($\times 10^{-3}$)
				Y_0 ($\times 10^{-3}$ Mho)	n		Y_0 ($\times 10^{-3}$ Mho)	n		Y_0 ($\times 10^{-3}$ Mho)	n	
m-CCO	260	4.94	-	-	-	3.10	45.0	0.55	15.8	6.6	0.97	2.24
m-CCO-NB	325	4.99	-	-	-	0.75	43.0	0.63	18.2	3.8	0.95	2.32
m-NCO	212	4.96	-	-	-	1.07	37.8	0.53	9.1	3.4	1.00	10.27
m-NCO-NB	269	4.97	0.31	31.6	0.44	0.71	16.8	0.66	22.4	4.4	0.93	4.52
m-ZCO	246	3.07	2.26	0.5	0.33	1.43	21.3	0.62	9.7	2.9	0.95	0.80
m-ZCO-NB	213	5.03	0.26	47.0	0.39	2.55	15.1	0.66	8.4	3.5	0.97	0.55

Table S13. Electrochemical characteristics of the prepared samples, measured on GC RDE electrode, in the ORR experiments.

Sample	E [d] (V *vs*. RHE)	$E_{½}$ [e] (V *vs*. RHE)	j_L [f] ($mA\cdot cm^{-2}$)	Tafel slope ($mV\cdot dec^{-1}$)	C_{dl} (μF)	ECSA [g] (cm^2)	S_{BET} [h] (cm^2)	ECSA/S_{BET}	e^- number [i] involving in ORR
m-CCO	0.641	0.664	4.8	100.3	850	21.25	55.5	0.38	3.73
m-CCO-NB	0.619	0.633	5.2	109.2	950	23.75	53.5	0.44	3.77
m-NCO	0.720	0.740	5.1	86.9	850	21.25	60.5	0.35	3.78
m-NCO-NB	0.743	0.745	5.7	89.4	1100	27.50	65.5	0.42	3.84
m-ZCO	0.707	0.722	5.2	81.7	1100	27.50	52.5	0.52	3.68
m-ZCO-NB	0.712	0.718	5.6	79.9	850	21.25	54.0	0.39	3.98

[d] Electrode potential at current density = -3.0 $mA\cdot cm^{-2}$.[23]

[e] Electrode potential at half current maximum.

[f] Diffusion-limiting current density.

[g] ECSA = $C_{dl} \times C_s$; C_s = 40 $\mu F\cdot cm^{-2}$.[24]

[h] Effective S_{BET} corresponding the catalyst loaded on the GC RDE electrode.

[i] Current on RDE electrode were subtracted by the current measured in N_2-saturated 0.1 M KOH solution.

References

(1) Puska, M. J.; Nieminen, R. M. Defect Spectroscopy with Positrons: A General Calculational Method. *J. Phys. F: Met. Phys.* **1983**, *13* (2), 333-346.

(2) Barbiellini, B.; Puska, M. J.; Korhonen, T.; Harju, A.; Torsti, T.; Nieminen, R. M. Calculation of Positron States and Annihilation in Solids: A Density-Gradient-Correction Scheme. *Phys. Rev. B* **1996**, *53* (24), 16201-16213.

(3) Bredar, A. R. C.; Chown, A. L.; Burton, A. R.; Farnum, B. H. Electrochemical Impedance Spectroscopy of Metal Oxide Electrodes for Energy Applications. *ACS Appl. Energy Mater.* **2020**, *3* (1), 66-98.

(4) Ding, R.; Qi, L.; Jia, M.; Wang, H. Simple Hydrothermal Synthesis of Mesoporous Spinel $NiCo_2O_4$ Nanoparticles and Their Catalytic Behavior in CH_3OH Electro-Oxidation and H_2O_2 Electro-Reduction. *Catal. Sci. Technol.* **2013**, *3* (12), 3207-3215.

(5) Li, G.; Anderson, L.; Chen, Y.; Pan, M.; Chuang, P.-Y. A. New Insights into Evaluating Catalyst Activity and Stability for Oxygen Evolution Reactions in Alkaline Media. *Sustainable Energy Fuels* **2018**, *2* (1), 237-251.

(6) Wang, J.; Fan, S.; Luan, Y.; Tang, J.; Jin, Z.; Yang, M.; Lu, Y. Ultrathin Mesoporous $NiCo_2O_4$ Nanosheets as an Efficient and Reusable Catalyst for Benzylic Oxidation. *RSC Adv.* **2015**, *5* (4), 2405-2410.

(7) Zou, R.; Xu, K.; Wang, T.; He, G.; Liu, Q.; Liu, X.; Zhang, Z.; Hu, J. Chain-Like $NiCo_2O_4$ Nanowires with Different Exposed Reactive Planes for High-Performance Supercapacitors. *J. Mater. Chem. A* **2013**, *1* (30), 8560-8566.

(8) Rong, H.; Chen, T.; Shi, R.; Zhang, Y.; Wang, Z. Hierarchical $NiCo_2O_4$@$NiCo_2S_4$ Nanocomposite on Ni Foam as an Electrode for Hybrid Supercapacitors. *ACS Omega* **2018**, *3* (5), 5634-5642.

(9) Xie, S.; Liu, Y.; Deng, J.; Yang, J.; Zhao, X.; Han, Z.; Zhang, K.; Dai, H. Insights into the Active Sites of Ordered Mesoporous Cobalt Oxide Catalysts for the Total Oxidation of O-Xylene. *J. Catal.* **2017**, *352*, 282-292.

(10) Wei, R.; Fang, M.; Dong, G.; Lan, C.; Shu, L.; Zhang, H.; Bu, X.; Ho, J. C. High-Index Faceted Porous Co_3O_4 Nanosheets with Oxygen Vacancies for Highly Efficient Water Oxidation. *ACS Appl. Mater. Interfaces* **2018**, *10* (8), 7079-7086.

(11) Ortiz-Quiñonez, J.-L.; Pal, U. Borohydride-Assisted Surface Activation of Co_3O_4/$CoFe_2O_4$ Composite and Its Catalytic Activity for 4-Nitrophenol Reduction. *ACS Omega* **2019**, *4* (6), 10129-10139.

(12) Kerber, S.; Bruckner, J.; Wozniak, K.; Seal, S.; Hardcastle, S.; Barr, T. The Nature of Hydrogen in X-Ray Photoelectron Spectroscopy: General Patterns from Hydroxides to Hydrogen Bonding. *J. Vac. Sci. Technol. A* **1996**, *14* (3), 1314-1320.

(13) Liu, S.; Ni, D.; Li, H.-F.; Hui, K. N.; Ouyang, C.-Y.; Jun, S. C. Effect of Cation Substitution on the Pseudocapacitive Performance of Spinel Cobaltite MCo_2O_4 (M= Mn, Ni, Cu, and Co). *J. Mater. Chem. A* **2018**, *6* (23), 10674-10685.

(14) Cheng, H.; Su, Y.-Z.; Kuang, P.-Y.; Chen, G.-F.; Liu, Z.-Q. Hierarchical $NiCo_2O_4$ Nanosheet-Decorated Carbon Nanotubes Towards Highly Efficient Electrocatalyst for Water Oxidation. *J. Mater. Chem. A* **2015**, *3* (38), 19314-19321.

(15) Hung, T.-F.; Mohamed, S. G.; Shen, C.-C.; Tsai, Y.-Q.; Chang, W.-S.; Liu, R.-S. Mesoporous $ZnCo_2O_4$ Nanoflakes with Bifunctional Electrocatalytic Activities toward Efficiencies of Rechargeable Lithium–Oxygen Batteries in Aprotic Media. *Nanoscale* **2013**, *5* (24), 12115-12119.

(16) Jung, S.; McCrory, C. C. L.; Ferrer, I. M.; Peters, J. C.; Jaramillo, T. F. Benchmarking Nanoparticulate Metal Oxide Electrocatalysts for the Alkaline Water Oxidation Reaction. *J. Mater. Chem. A* **2016**, *4* (8), 3068-3076.

(17) Mai, Z.; Duan, W.; Wang, K.; Tang, Z.; Chen, S. Integrating $ZnCo_2O_4$ Submicro/Nanospheres with Co_xSe_y Nanosheets for the Oxygen Evolution Reaction and Zinc–Air Batteries. *Sustainable Energy Fuels* **2020**, *4* (5), 2184-2191.

(18) Mugheri, A. Q.; Tahira, A.; Aftab, U.; Bhatti, A. L.; Memon, N. N.; Memon, J.-u.-R.; Abro, M. I.; Shah, A. A.; Willander, M.; Hullio, A. A.; Ibupoto, Z. H. Efficient Tri-Metallic Oxides $NiCo_2O_4$/CuO for the Oxygen Evolution Reaction. *RSC Adv.* **2019**, *9* (72), 42387-42394.

(19) Priamushko, T.; Budiyanto, E.; Eshraghi, N.; Weidenthaler, C.; Kahr, J.; Jahn, M.; Tuysuz, H.; Kleitz, F. Incorporation of Cu/Ni in Ordered Mesoporous Co-Based Spinels to Facilitate Oxygen Evolution and Reduction Reactions in Alkaline Media and Aprotic Li-O_2 Batteries. *ChemSusChem* **2022**, *15* (5), No. e202102404.

(20) Zhang, J.; Zhang, D.; Yang, Y.; Ma, J.; Cui, S.; Li, Y.; Yuan, B. Facile Synthesis of $ZnCo_2O_4$ Mesoporous Structures with Enhanced Electrocatalytic Oxygen Evolution Reaction Properties. *RSC Adv.* **2016**, *6* (95), 92699-92704.

(21) Wang, X. T.; Ouyang, T.; Wang, L.; Zhong, J. H.; Liu, Z. Q. Surface Reorganization on Electrochemically-Induced Zn-Ni-Co Spinel Oxides for Enhanced Oxygen Electrocatalysis. *Angew. Chem. Int. Ed.* **2020**, *59* (16), 6492-6499.

(22) Devaguptapu, S. V.; Hwang, S.; Karakalos, S.; Zhao, S.; Gupta, S.; Su, D.; Xu, H.; Wu, G. Morphology Control of Carbon-Free Spinel $NiCo_2O_4$ Catalysts for Enhanced Bifunctional Oxygen Reduction and Evolution in Alkaline Media. *ACS Appl. Mater. Interfaces* **2017**, *9* (51), 44567-44578.

(23) Gorlin, Y.; Jaramillo, T. F. A Bifunctional Nonprecious Metal Catalyst for Oxygen Reduction and Water Oxidation. *J. Am. Chem. Soc.* **2010**, *132* (39), 13612-13614.

(24) Yu, M.; Moon, G.; Bill, E.; Tüysüz, H. Optimizing Ni–Fe Oxide Electrocatalysts for Oxygen Evolution Reaction by Using Hard Templating as a Toolbox. *ACS Appl. Energy Mater.* **2019**, *2* (2), 1199-1209.
